# Phenotypic and genetic analysis of a wellbeing factor score in the UK Biobank and the impact of childhood maltreatment and psychiatric illness

**DOI:** 10.1038/s41398-022-01874-5

**Published:** 2022-03-19

**Authors:** Javad Jamshidi, Peter R. Schofield, Justine M. Gatt, Janice M. Fullerton

**Affiliations:** 1 Neuroscience Research Australia, Sydney, NSW Australia; 2 School of Psychology, University of New South Wales, Sydney, NSW Australia; 3 School of Medical Sciences, University of New South Wales, Sydney, NSW Australia

**Keywords:** Genomics, Human behaviour

## Abstract

Wellbeing is an important aspect of mental health that is moderately heritable. Specific wellbeing-related variants have been identified via GWAS meta-analysis of individual questionnaire items. However, a multi-item within-subject index score has potential to capture greater heritability, enabling improved delineation of genetic and phenotypic relationships across traits and exposures that are not possible on aggregate-data. This research employed data from the UK Biobank resource, and a wellbeing index score was derived from indices of happiness and satisfaction with family/friendship/finances/health, using principal component analysis. GWAS was performed in Caucasian participants (*N* = 129,237) using the derived wellbeing index, followed by polygenic profiling (independent sample; *N* = 23,703). The wellbeing index, its subcomponents, and negative indicators of mental health were compared via phenotypic and genetic correlations, and relationships with psychiatric disorders examined. Lastly, the impact of childhood maltreatment on wellbeing was investigated. Five independent genome-wide significant loci for wellbeing were identified. The wellbeing index had SNP-heritability of ~8.6%, and stronger phenotypic and genetic correlations with its subcomponents (0.55–0.77) than mental health phenotypes (−0.21 to −0.39). The wellbeing score was lower in participants reporting various psychiatric disorders compared to the total sample. Childhood maltreatment exposure was also associated with reduced wellbeing, and a moderate genetic correlation (*r*_g_ = ~−0.56) suggests an overlap in heritability of maltreatment with wellbeing. Thus, wellbeing is negatively associated with both psychiatric disorders and childhood maltreatment. Although notable limitations, biases and assumptions are discussed, this within-cohort study aids the delineation of relationships between a quantitative wellbeing index and indices of mental health and early maltreatment.

## Introduction

Wellbeing is an important aspect of mental health, and is defined by the core concepts of happiness, life satisfaction and optimal psychological functioning [1]. This concept of wellbeing is consistent with the World Health Organisation definition of health: “a state of complete physical, mental and social wellbeing and not merely the absence of disease or infirmity”.

Wellbeing is defined by two core concepts: (1) subjective wellbeing (also referred to as ‘hedonia’), consisting of positive affect and satisfaction with life, and (2) psychological wellbeing (also referred to as ‘eudaimonia’), consisting of attributes that enable realisation of human potential including autonomy, mastery and life purpose [2]. Various questionnaires measure wellbeing [3, 4], but questionnaire items usually load onto a common wellbeing factor [5]. Indeed, we showed that a composite measure encapsulating elements of both subjective and psychological wellbeing, is more heritable and stable longitudinally than wellbeing indices derived from single-item questions [6].

Wellbeing is moderately heritable, with estimates from twin studies ranging from 17 to 67% [reviewed in Bartels [7]], leading to efforts to elucidate the specific genetic signatures (particularly Single Nucleotide Polymorphisms (SNPs)) underlying this trait [8, 9]. However, SNP-heritability estimated from genome-wide association studies (GWAS) are only ~4–6.4% [8, 9], thus new approaches are required to explain a greater proportion of trait heritability.

To improve power for gene discovery, analytic approaches have included meta-analyses of independent GWAS from different cohorts of the component traits which underlie subjective wellbeing [8]. As wellbeing is genetically and phenotypically correlated to negative mental health constructs including neuroticism and depressive symptoms [8, 10], other analytic approaches have used multivariate GWAS meta-analysis to include additional correlated phenotypes, as part of a ‘wellbeing spectrum’ [10, 11] that spans correlated traits such as loneliness and self-rated health [12]. However, broadening the spectrum to include negative health outcomes as a continuum may diminish the detection of unique genetic signals specifically relating to wellbeing. For example, previous studies have shown negative genetic correlations between subjective wellbeing and anxiety, depression, schizophrenia and bipolar disorder (ranging from −0.73 to −0.22) [13], suggesting different proportions of common and unique genetic factors across the spectrum of negative health outcomes. By separating the constructs and quantifying the influence of psychiatric disorders on wellbeing, one can subsequently examine the impact of environmental exposures known to predict higher risk for certain disorders. Furthermore, as no phenotype is derivable using meta-analytic genetic approaches, one cannot use this method to examine the phenotypic properties of wellbeing – this includes examining phenotypic correlations, quantifying the influence of psychiatric disorders, and environmental exposures. Therefore, a wellbeing score composed of multiple relevant unique components would facilitate detailed exploration of both genetic and phenotypic attributes of wellbeing.

One potentially important environmental influence on wellbeing is childhood maltreatment, for which a negative impact on wellbeing has been demonstrated in adulthood [14, 15]. Furthermore, recent evidence suggests that there is a genetic contribution to childhood maltreatment through gene–environment correlations [16, 17], suggesting that childhood maltreatment may influence wellbeing through both genetic and environmental processes, a relationship that has not hitherto been explored.

Here, building on our previous work [6], we hypothesise that a continuous wellbeing index – derived from multiple measures of wellbeing available in the UK Biobank – is more heritable than single-item measures, and can be used to explore both phenotypic and genetic correlations with related clinical features and environmental exposures. The availability of genotype data from UK Biobank participants provides the opportunity to use various genetic techniques, including gene discovery, genetic correlation analyses and exploration of predictive power of polygenic scores. Herein, we use five approaches to evaluate the wellbeing index. First, phenotypic correlations between the wellbeing index, its subcomponents and related negative mental health constructs are measured. Second, we perform discovery GWAS for the wellbeing index and functional analysis of identified genetic signals. Third, genetic correlations of wellbeing-related phenotypes are compared, head-to-head within the same sample. We also examine genetic correlations with several psychiatric illnesses and quantify the effect of specific psychiatric diagnoses on the wellbeing index. Fourth, we assess the predictive power of wellbeing index polygenic scores in an independent UK Biobank-derived sample. Finally, we evaluate the effect of childhood maltreatment exposure on the wellbeing index and examine their genetic correlations.

## Materials and methods

### Participants

The UK Biobank is a population-based cohort with >500,000 participants from the United Kingdom, aged 40–69 years when recruited at baseline between 2006 and 2010, of predominantly British-Angloceltic ancestry [18]. Potentially eligible participants were identified from population-based registers and invited to participate. Due to the broad scope of the resource, the baseline questionnaire was designed to include questionnaire items with reliability and validity that relate to outcomes of public health importance. The UK Biobank received approval by the National Health Service National Research Ethics Service (11/NW/0382) and participants provided signed informed consent. The current project was approved by UK Biobank Data Access Committee (58534), with approval by University of New South Wales Human Research Advisory Panel (HC200191) for data analysis.

### Defining wellbeing

We reviewed all UK Biobank questionnaire items and selected five wellbeing-related items from the baseline questionnaire, including general happiness and satisfaction with family, friendship, health and financial situation (Data-Fields:4526, :4559, :4570, :4581, :4548, respectively). Responses were on a 6-point Likert scale, related to general life experiences (not a defined timescale) and were reverse-scored. Principal component analysis was performed in SPSSv25, and a factor score created, herein referred to as the “wellbeing index”. To evaluate goodness of model fit, we employed confirmatory factor analysis in the validation dataset using R package *lavvan*. The internal consistency of the wellbeing index was tested using Cronbach’s Alpha. Additional details and analysis workflow are in Supplementary Methods (Fig. S1).

### Discovery and confirmatory samples

To define the discovery sample, we used UK Biobank genetic data (March-2018 release), comprising 488,000 individuals genotyped using Affymetrix UK BiLEVE Axiom or UK Biobank Axiom arrays. Further information on the cohort, genotyping, imputation, and quality control (QC), is available elsewhere [18, 19]. Briefly, participants were removed who withdrew consent, reported non-Caucasian ancestry (Data-Field:22006), had >10% genotype missingness or QC failure (Data-Field:22051), were on genomic analysis exclusion list (Data-Field:22010), had gender mismatch (self-report vs. genotype-derived), sex chromosome aneuploidy or heterozygosity outliers (Data-Field:22027).

Participants meeting genotype QC criteria, with complete phenotype data for the five core wellbeing questions, were *N* = 129,237. Analysis of other mental health indicators was limited to this sample, but due to missingness of some phenotypes, sample size varies slightly.

An independent sample from UK Biobank was defined (*N* = 23,703; see Supplementary Methods) for confirmatory factor analysis, and to evaluate predictive power of polygenic scores from discovery GWAS.

### Negative mental health indicators

Four negative mental health indicators were selected as outcome comparators for the wellbeing index score. These included measures of loneliness, neuroticism, depressive symptoms and whether the participant had ever seen a psychiatrist or GP for nerves, anxiety, tension or depression (Data-Fields provided in Table S1). Among these measures, loneliness and neuroticism ask about current experiences whereas the depressive symptoms and “seen a psychiatrist or GP” ask about lifetime experiences. Variable coding is described in Supplementary Methods.

### Childhood maltreatment

Five items related to childhood maltreatment were identified, including emotional or physical neglect, and physical, emotional, or sexual abuse [Data-Field:20487-20490], and responses were on a 5-point Likert scale (see Supplemental Material). These exposures are collectively referred to as “maltreatment”, henceforth. First, the impact of each type of childhood maltreatment on the wellbeing index was examined as categorical variables, relating to the frequency of maltreatment experience. Then to evaluate the cumulative impact of maltreatment exposure, each item was dichotomised as “exposed” or “not-exposed” [see Table S2 for details], and a *sum-score* was created by summing across four maltreatment types [ranging from 0 to 4; excluding physical neglect due to identified coding issue (see Supplemental Methods for details)]. There were 45,723 participants with non-missing childhood maltreatment sum-score and wellbeing index.

Mean differences in wellbeing index were examined using Kruskal-Wallis (multi-category), and Wilcoxon (two-category) tests. Simple and multiple linear regression models, adjusting for age, age-squared, sex and Townsend Deprivation Index [Data-Field:189], examined how childhood maltreatment (or exposure sum-score) impacts wellbeing. The regression standardised estimate was considered the magnitude of effect of the dependant variable.

### Psychiatric illnesses

The question “Have you been diagnosed with one or more of the following mental health problems by a professional, even if you don’t have it currently?” (Data-Field:20544) was used as the indicator of a lifetime psychiatric diagnosis. “Prefer not to answer” responses were coded as missing. As participants could select ≥1 of 16 listed diagnoses, representation in each mental health category was not mutually exclusive.

### Phenotypic correlations *(r*_p_*)*

The *correlation* (v0.6.0) R package was employed to examine phenotypic correlations *(r*_p_*)*, employing different types of correlations based on data type: Spearman’s rank for continuous/ordinal variables, Tetrachoric for binary, and Point-biserial for continuous/ordinal versus binary. Analyses were limited to participants with no missing phenotype data to enable head-to-head comparisons using the same sample size across all measures (*N* = 103,373; due to missing data in negative mental health phenotypes). The significance threshold for *r*_p_ was set at *p* < 0.001, using a Bonferroni correction for 45 tests (*α* = 0.05/45).

### Genetic association and SNP-heritability

To perform GWAS, we employed BOLT-LMM v2.3.4 [20]. Analysis employed imputed genotypes in BGENv.1.2 format (v3) and incorporated a Bayesian linear mixed effects model (LMM), accounting for population structure and sample relatedness using a genetic relatedness matrix, plus relevant covariates (sex, age, age-squared, genotype array, 20 PCs). BOLT-LMM automatically filters SNPs and individuals with >10% missing, and further filtering of SNPs with MAF < 0.01, INFO < 0.8 and Hardy-Weinberg Equilibrium *p* < 1 × 10^−6^, left ~8,068,119 SNPs for analysis. Results from the standard infinitesimal mixed-model association were employed for downstream analysis.

Functional Mapping and Annotation of GWAS (FUMA) [21] was employed to link candidate SNPs to genes and perform functional analysis (see Supplementary Methods).

SNP-heritability calculations employed Linkage Disequilibrium Score Regression (LDSC) [22], utilising GWAS summary statistics for each target phenotype derived from the same discovery sample. We used the LDSC method to estimate heritability and genetic correlations because it is a widely adopted method employed by most previous studies, hence ensuring more comparable associations. However, we acknowledge that this method has several assumptions that could limit the accuracy of estimates [22, 23], which are discussed later.

### Genetic correlations (*r*_g_)

LDSC [22] was used to estimate genetic correlations (*r*_g_) between the wellbeing index, its five subcomponents, four negative indicators of mental health, and childhood maltreatment using GWAS summary statistics generated herein.

Published summary statistics of additional wellbeing-related traits were used to estimate *r*_g_ with the derived wellbeing index phenotype, including: positive affect, life satisfaction, wellbeing spectrum, [10] subjective wellbeing [8], conscientiousness [24], extraversion [25], neuroticism [26], depressive symptoms [8], loneliness [27] and body mass index (BMI) [28].

In addition, *r*_g_ with major psychiatric conditions were examined using published disease-specific summary statistics, including: major depressive disorder (MDD) [29], bipolar disorder (BIP) [30], schizophrenia (SCZ) [31], attention deficit/hyperactivity disorder (ADHD) [32], autism spectrum disorder (ASD) [33], obsessive-compulsive disorder (OCD) [34] and post-traumatic stress disorder (PTSD) [35]. We included HapMap3 SNPs with MAF > 0.01 and excluded the MHC region for *r*_g_ estimations. The significance threshold for *r*_g_ after Bonferroni correction for 72 tests (*α* = 0.05/72) was *p* < 6.94 × 10^−4^.

### Polygenic scores

Polygenic scores (PGS) were constructed in the independent confirmatory cohort using PRS-CS software [36], employing summary statistics of the wellbeing index GWAS. We tested the predictive power of PGS in the confirmatory sample for the wellbeing index, its subcomponents, and negative mental health indicators using regression models, including relevant covariates (age, age-squared, sex, genotyping array, 10 PCs) in all models. An incremental R^2^, defined as the difference between the R^2^ from the full-model and the model without PGS as a predictor, was reported as predictive power. For binary variables (loneliness, depressive symptoms and “seen GP or psychiatrist”) a logistic regression was used instead of linear regression, and Nagelkerke R^2^ calculated using the R package *fmsb* (v0.7.0). A 95% confidence interval around the R^2^ was calculated using the R package *psychometric* (v2.2).

## Results

### Participant demographics

The discovery cohort comprised 129,237 participants (57.9% female) who were of British-white ancestry and had no missing phenotypes for the five core wellbeing questions. The age range was 40-70 years (mean ± SD = 57.3 ± 8.0), and 31.9% reported having college education. The data-field IDs and description of study variables in the discovery sample is presented in Table S1.

The confirmatory sample comprised 23,703 participants (51.3% female), who were genetically unrelated to the discovery sample but with identical inclusion criteria, and were ~7 years older (mean ± SD = 64.5 ± 7.7, range 46–82 years) than discovery cohort participants.

### The wellbeing index and phenotypic correlations

Principal component analysis in the discovery sample indicated the first component explained 47.48% of the variance of the five questions (eigenvalue = 2.374) (Fig. S2), and was extracted using regression method as the wellbeing index score. The wellbeing index was approximately normally distributed with an extended left tail (skewness = −0.373, kurtosis=4.119) (Fig. S3A). Females had a slightly higher mean score than males (0.029 vs −0.034 respectively; Wilcoxon *p* < 2.2 × 10^−16^; Fig. S3B). The wellbeing index had a good internal consistency with Cronbach’s Alpha of 0.71. Confirmatory factor analysis in the independent sample showed a good model fit, loading onto a single factor (CFI = 0.98, TLI = 0.97, RMSEA = 0.047, SRMR = 0.034).

**Fig. 1:**
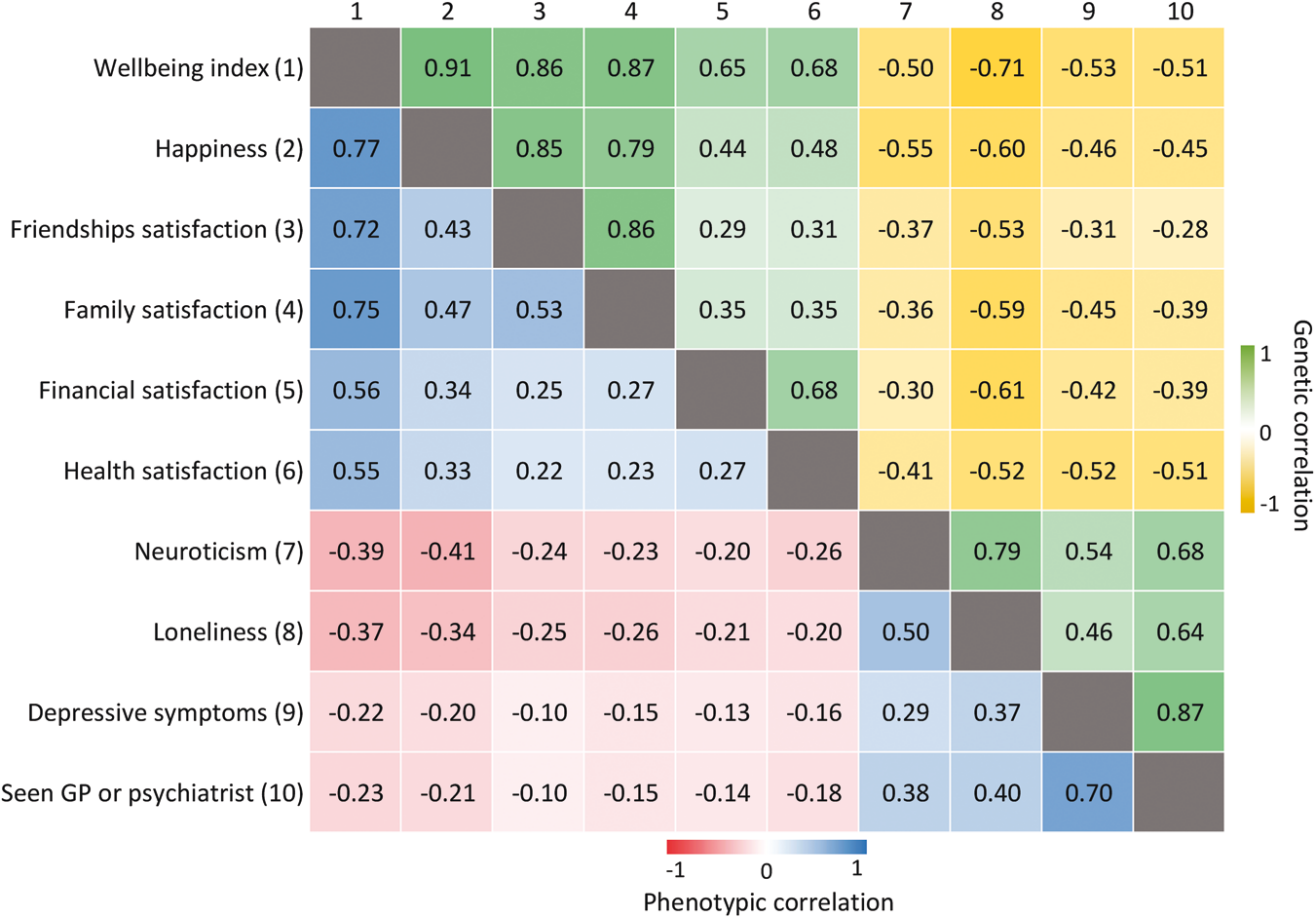
Phenotypic and genetic correlations between the wellbeing index score, its subcomponents and negative mental health indicators in UK Biobank. All correlations reported here are statistically significant after Bonferroni correction for multiple testing, although many have low (*r* = 0.3–0.5) to moderate (*r* = 0.5–0.7) effect size. Manhattan plots for each GWAS are provided in Figure S6.

The wellbeing index had the strongest *r*_p_ with the subcomponent happiness (*r*_p_ = 0.77), and the weakest with health satisfaction (*r*_p_ = 0.55). From the negative mental health indicators, the wellbeing index had the strongest *r*_p_ with neuroticism (*r*_p_ = −0.39) and weakest with depressive symptoms (*r*_p_ = −0.22) (Fig. 1).

### GWAS findings for wellbeing index

**Fig. 2:**
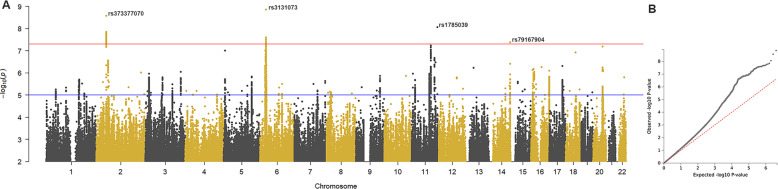
Genome-wide association analysis of the wellbeing index score in UK Biobank discovery sample. **A** Manhattan plot representing association at 8,068,119 SNPs across the genome, with chromosome and base pair position is on x-axis and negative logarithm of the p-value from infinitesimal model is on y-axis. The red line indicates the genome-wide significance threshold of *p* < 5×10^−8^. **B** Quantile-quantile plot showing inflation of observed associations over that expected under null (λ_GC_ = 1.2005, mean χ^2^ = 1.2171, LD score regression intercept=1.005, Total Observed scale *h*^2^ = 0.0857 ± 0.005).

Forty-two SNPs exceeded genome-wide significance for association from wellbeing index GWAS (*p* < 5 × 10^–8^) (Fig. 2), which represented five independent loci (Table S3 and Fig. S4).

Candidate genes at each locus were mapped in FUMA using positional (*n* = 44 genes), eQTL (*n* = 27) and chromatin interaction mapping (*n* = 62) modules (total *n* = 101) (Table S4). The GWAS catalogue in FUMA identified signal overlaps amongst SNPs in linkage disequilibrium with wellbeing index and related traits; on chromosome 2, *FSHR* was associated with hedonic wellbeing [9] and both loci on chromosome 6 – the first associated with many related conditions including positive-affect, wellbeing spectrum [9], and depression [8, 29], and the second centred around *TRIM26* associated with autism and schizophrenia [37, 38] (Table S5).

Tissue-specific enrichment of wellbeing index GWAS genes in MAGMA (implemented in FUMA) [39] showed significant enrichment in brain tissues, particularly cerebellum and frontal cortex (Fig. S5). Manhattan plots for GWAS of wellbeing subcomponents and related traits are provided (Fig. S6a–j).

### Heritability and genetic correlations

For wellbeing and its subcomponents, the wellbeing index had the highest SNP-heritability (*h*^2^ = 8.6%, SE = 0.005), although it was not significantly different from health satisfaction (*h*^2^ = 7.8%, SE = 0.005). Other subcomponents had similar SNP-heritability (*h*^2^ = 6.2–5.5%, SE = 0.005) (Fig. S7).

Generally, the *r*_g_ between traits were stronger than their *r*_p_ (Fig. 1), and *r*_g_ and *r*_p_ were strongly correlated (*r* = 0.97). Amongst the subcomponents (Fig. S6a–e), wellbeing index had the strongest relationship with happiness (*r*_g_ = 0.91, SE = 0.013). Across the negative mental health indicators (Fig. S6f-j), wellbeing index had the strongest relationship with loneliness (*r*_g_ = −0.71, SE = 0.041).

**Fig. 3:**
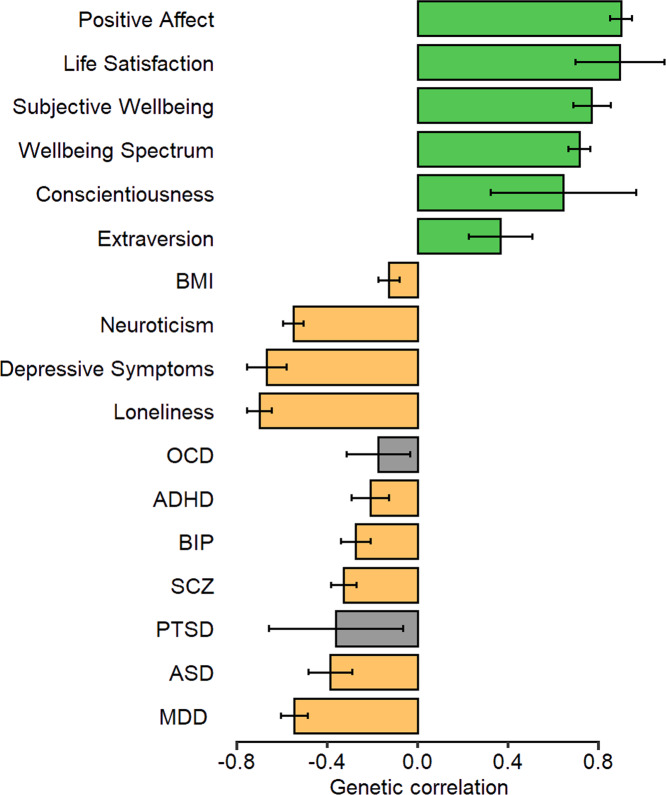
Genetic correlation (*r*_g_) between the wellbeing index score and published GWAS of relevant phenotypes and psychiatric disorders. Positively and negatively correlated phenotypes and major psychiatric illnesses were examined using Linkage Disequilibrium Score Regression (LDSC). Published wellbeing-related summary statistics from independent studies included: positive affect, life satisfaction, wellbeing spectrum [10], subjective wellbeing [8], conscientiousness [24], extraversion [25], neuroticism [26], depressive symptoms [8], loneliness [27] and body mass index (BMI) [28]. Published disease-specific summary statistics from independent studies included: major depressive disorder (MDD) [29], bipolar disorder (BIP) [30], schizophrenia (SCZ) [31], attention deficit/hyperactivity disorder (ADHD) [32], autism spectrum disorder (ASD) [33], obsessive-compulsive disorder (OCD) [34] and post-traumatic stress disorder (PTSD) [35]. Error bars represent 95% confidence intervals (95% CI). Green bars indicate positive *r*_g_, orange bars indicate negative *r*_g_ and grey bars indicate traits with non-significant genetic correlation after Bonferroni correction (*p* > 6.94 × 10^−4^).

Using published summary statistics, the *r*_g_ between wellbeing index and other positively and negatively related phenotypes showed significant correlation for all tested traits after multiple-testing correction. Except for OCD and PTSD, all psychiatric conditions had a significant negative relationship with wellbeing index – the strongest effect was for MDD (*r*_g_ = −0.55, SE = 0.03) (Fig. 3).

### Polygenic score analysis

**Fig. 4:**
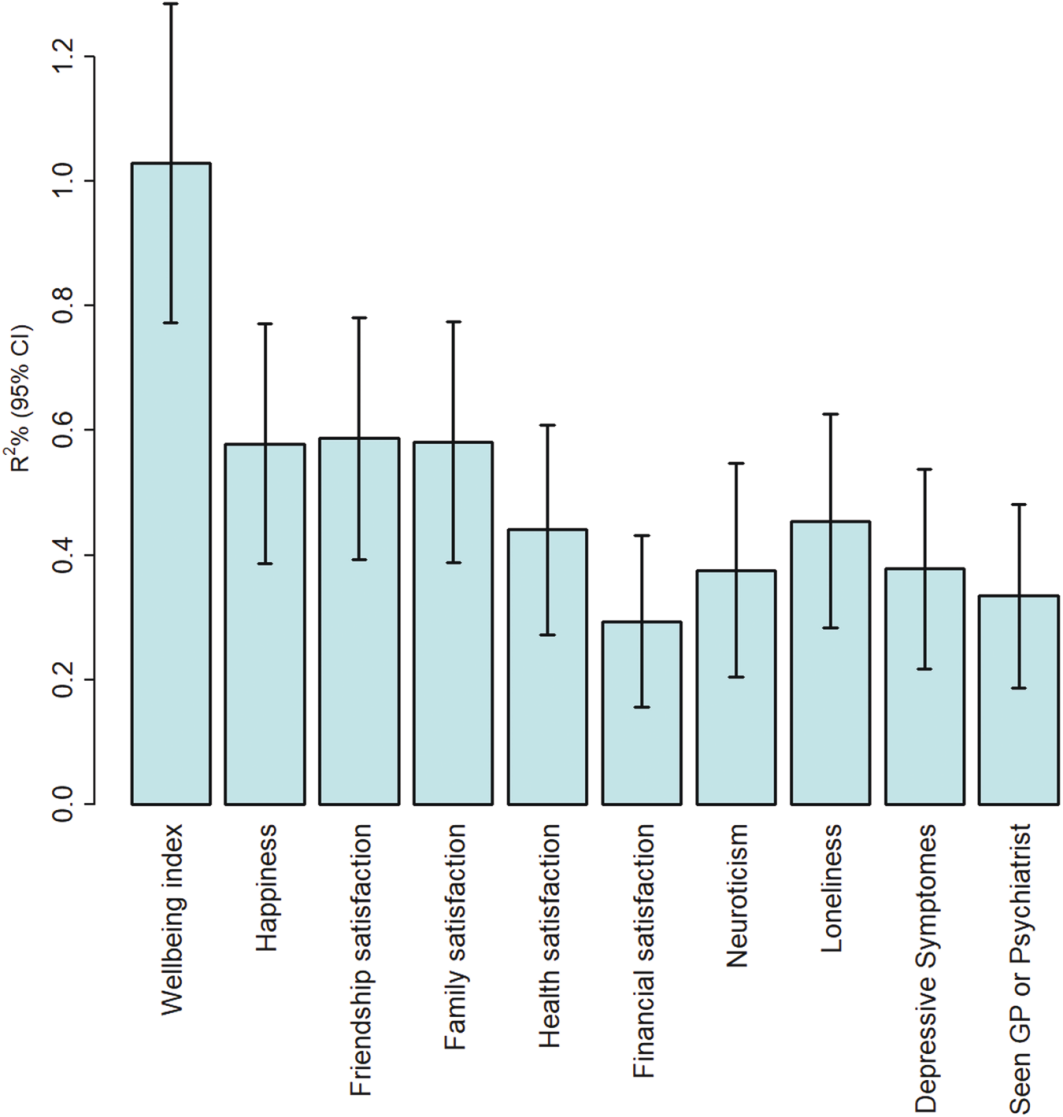
Variance explained by polygenic scores derived from the wellbeing index discovery GWAS in the replication cohort for wellbeing index score, its subcomponents and related negative mental health indicators. The y-axis shows the incremental R^2^% and for binary variables (Loneliness, Depressive symptoms, and Seen GP or Psychiatrist) Nagelkerke R^2^. Error bars are lower and upper bound of 95% confidence intervals. While variance explained was <1%, *P*-values were all highly significant (*p* = 3.55 × 10^−14^ – 5.68 × 10^−56^).

In the replication sample, the wellbeing index PGS explained a small but significant portion of variance in all traits examined, with highest predictive power for the wellbeing index score itself (*R*^2^ = 0.01, *p* = 5.68 × 10^−56^), and the lowest for financial satisfaction (*R*^2^ = 0.003, *p* = 2.77 × 10^−17^). Amongst negative mental health indices, the best predictive power of wellbeing PGS was for loneliness (Nagelkerke *R*^2^ = 0.004, *p* = 2.93 × 10^−14^; Fig. 4).

### Childhood maltreatment and wellbeing index

For all types of childhood maltreatment, the wellbeing index showed a stepwise reduction with increased frequency of maltreatment exposure (Fig. S8) – noting the potential data coding issue identified impacting physical neglect (Data-Field:20491) which indicated misinterpretation of the “never” response category, resulting in this variable’s exclusion (see Supplemental Methods).

**Fig. 5:**
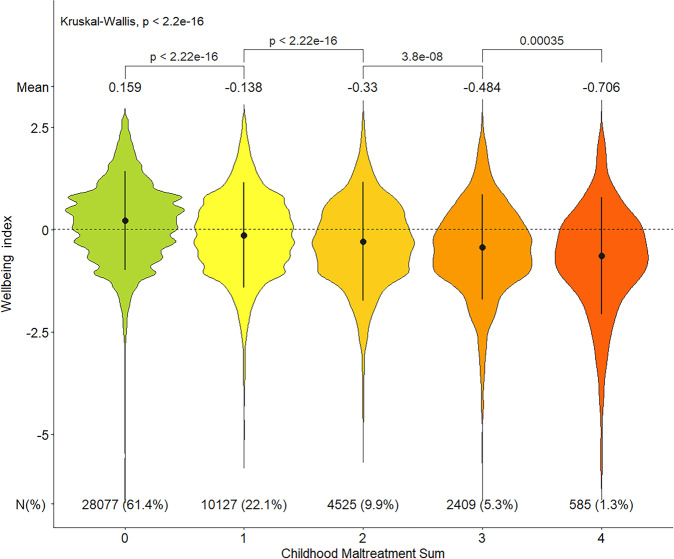
The impact of multiple childhood maltreatment exposure on the wellbeing index. The x-axis shows the childhood maltreatment sum score which represents number of childhood traumas in each group (0 = no trauma, 4 = experienced all four types of traumas). The y-axis is the wellbeing index *z*-score. The mean of wellbeing index z-score for each group is shown above each violin plot. The mean difference in wellbeing index between all categories was examined using Kruskal–Wallis test (*p* < 2.2 × 10^−16^). The pairwise mean difference between two adjacent groups employed the Wilcoxon test and the *p*-values are presented. The interquartile range is represented by vertical black lines inside the violin plots, and the dotted horizontal line is the median wellbeing index score in the sample (*n* = 45,723). The number of participants in each group is shown at the bottom of each category.

Among participants reporting any childhood maltreatment, 43% reported multiple exposure types. The effect was dose dependant: by accumulation of maltreatment types, the mean wellbeing score decreased by about 0.2 for each additional maltreatment (Fig. 5). All types of childhood maltreatment exposure were significant in linear regression models predicting wellbeing. In simple models (with one trauma type as the predictor), the highest effect estimate was for the maltreatment sum score (*β* = −0.206, SE = 0.005), although not significantly different from emotional neglect (*β* = −0.200, SE = 0.010) (Fig. S9, panel A). In multiple linear model (with all traumas as predictors), emotional neglect and abuse had larger effects than physical or sexual abuse (Fig. S9, panel B). In people with exposure to a single maltreatment type, the wellbeing index was most significantly influenced by emotional neglect (mean = −0.373; Fig. S10).

### Childhood maltreatment GWAS and genetic correlations

Three loci exceeded genome-wide significance for association with childhood maltreatment: chromosome 7 (rs1015511, *p* = 1.7 × 10^−12^), chromosome 15 (rs4702, *p* = 4.3 × 10^−12^) and chromosome 16 (rs2043596, *p* = 2.4 × 10^−8^) (Fig. S6J). There was a significant negative *r*_g_ between childhood maltreatment sum score and wellbeing index (*r*_g_ = −0.56, *p* = 8.21 × 10^−54^) and its components (*r*_g_ = −0.60 to −0.37), and a positive *r*_g_ with negative indicators of mental health (Fig. S11). The strongest negative *r*_g_ was for family satisfaction (*r*_g_ = −0.60, *p* = 2.61 × 10^−46^) and the strongest positive *r*_g_ for depressive symptoms (*r*_g_ = 0.57, *p* = 1.61 × 10^−37^).

### Psychiatric diagnoses and wellbeing

The mean wellbeing index *z*-score was negative for 16 self-reported psychiatric conditions (−1.66 to −0.22) (Fig. S12). Some psychiatric diagnoses were rare in the population, precluding formal statistical analysis. The most frequent psychiatric conditions – “depression” (*N* = 9,944; 7.7%) and “Anxiety, nerves or generalised anxiety disorder” (*N* = 6,508; 5%) were associated with lower mean wellbeing index *z*-score of −0.355 and −0.289, respectively. Of mental illness groups with >200 participants (>0.2% population frequency), the largest mean differences were with over-eating/binge-eating, social-anxiety/social-phobia, OCD, and bipolar-mania.

## Discussion

A wellbeing index score was constructed and evaluated phenotypically and genetically in the UK Biobank. The wellbeing index had moderate to high correlation with its subcomponents (*r*_g_ = 0.65–0.91, *r*_p_ = 0.55–0.77) and moderate to low negative correlation with adverse mental health indicators (*r*_g_ = −0.71 to −0.50, *r*_p_ = −0.39 to −0.22). The wellbeing GWAS had five genome-wide significant associated loci, with a relatively high SNP-heritability. Despite very small estimated effects on phenotypic variance, the wellbeing index PGS significantly predicted the wellbeing score and correlated items in an independent sample. Moreover, both childhood maltreatment and psychiatric illness were associated with lower wellbeing.

### Strengths and weaknesses

While previous meta-analyses of GWAS summary statistics for correlated phenotypes has significantly advanced our understanding of wellbeing genetics [8, 10, 11], these methods have limitations. First, employing genetic-based methods on aggregated data ignores clinical and genetic heterogeneity, and precludes detailed analysis of corresponding phenotypic environmental modifiers. Second, the SNP-heritability of the resulting phenotype is usually reduced due to heterogeneity in the phenotypes (e.g., SNP-heritability of “wellbeing spectrum” derived from multi-trait meta-analysis is only ~0.02 [10]). Herein, we used the same measures for all participants, making it possible to precisely examine *r*_p_ and *r*_g_ relationships. We demonstrated that SNP-heritability of the wellbeing index score was slightly higher than previously reported wellbeing SNP-heritability (*h*^2^ = 8.6% vs. ~4.0–6.4%) [8–10]. Thus, as covariance is greater due to elevated SNP-heritability, the wellbeing index improves reliability for genetic correlation analysis. However, while the present wellbeing index slightly increased SNP-heritability estimates compared to those previously reported, most of the causal variants for well-being were not discovered, and SNP-heritability remains much smaller than the heritability estimates derived from twin studies (phenotypic heritability of ~36% [7] vs. SNP-heritability of 8.6%). This ‘missing heritability’ highlights the inability of conventional genetic methods to uncover the complete variance of causal variants for complex traits such as wellbeing, which are likely the result of an intricate interaction of genes and environmental factors in the presence of heterogeneity, pleiotropy, and epistasis. The challenge to close the gap between twin and SNP-heritability estimates likely requires alternative analytic approaches and carefully phenotyped cohorts, with more delicate methods such as machine learning and incorporation of non-linear models of gene-gene and gene-environment effects [40, 41], which seem promising avenues that may provide better understanding of networks involving complex interrelated phenotypes [42].

Although mental illness can influence wellbeing, we defined wellbeing in the entire population – including those with a mental illness – to ensure that findings were closely representative of a general population, within inherent cohort selection biases [43]. While the mean wellbeing index in participants reporting a psychiatric condition was lower than the general population, some individuals in each mental illness group had a positive *z*-score, indicating that even within a background of mental illness an individual’s wellbeing can be positive – supporting the notion that wellbeing is not simply the absence of mental illness [44–46]. However, these results should be interpreted with caution, as psychiatric conditions herein were self-reported, not mutually exclusive (due to psychiatric comorbidities), and could have been current or historical, with variable impacts on current wellbeing. Finally, we cannot conclude causation or infer direction of effect between psychiatric illness and wellbeing, as effects are likely bidirectional. However, the negative *r*_g_ between wellbeing index and major psychiatric illnesses implies that relationships might be partly due to overlapping genes or gene-environmental correlations influencing both outcomes.

While the wellbeing index was derived from the baseline questionnaire, childhood maltreatment data was collected in the follow-up mental health assessment, 6–10 years later. We did not consider this a methodological concern given that the trauma questionnaire asked retrospectively about childhood events. Notably, we observed discrepancies relating to response endorsement for physical neglect – this potential issue has not, to our knowledge, been reported previously – hence we recommend caution with the use of Data-Field:20491, which was excluded herein. The stepwise decline in mean wellbeing index with increasing frequency of each individual type of maltreatment, as well as maltreatment accumulation, is consistent with existing evidence indicating that frequency and accumulation of different trauma types are key factors that can influence the way childhood maltreatment affects adults [15, 47, 48]. Furthermore, the largest effect on wellbeing index was for *emotional* rather than physical and sexual maltreatments – consistent with a recent report [49]. Collectively, emotional maltreatment appears to have profound consequences on wellbeing and mental health, potentially due to adverse developmental consequences in understanding and controlling emotions and cognitive development.

A strong correlation of *r*_g_ and *r*_p_ was found across all phenotypes (*r* = 0.97), with *r*_g_ consistently higher than *r*_p_ (e.g., loneliness and neuroticism *r*_g_ = 0.79, *r*_p_ = 0.50) – consistent with comparisons of genotype/phenotype correlations of other traits [50]. We note that the nature of *r*_p_ and *r*_g_ are somewhat distinct, with the former comparing individual-level data (and different variable types), and the latter comparing summary statistics across a population. Therefore, these findings should be interpreted with caution. A stronger *r*_g_ for wellbeing-related phenotypes has previously been reported [12], and a recent study observed that even phenotypically uncorrelated mental health profiles can be genetically correlated, suggesting either a genetic overlap that is distinct from clinical overlap, or unique environmental factors impact the phenotype in the presence of pleiotropy [51]. The stronger *r*_g_ between two traits may indicate that although shared genetic variants influence both phenotypes, environmental factors may impact them differently [as discussed [50]]. Amongst negative mental health indicators, loneliness had the strongest negative *r*_g_ with the wellbeing index, as well as its subcomponents. This further highlights the importance of loneliness in wellbeing [12], which despite a moderate to weak *r*_p_, shows strong *r*_g_ [52]. Interestingly, loneliness had the strongest *r*_g_ with the financial satisfaction subcomponent, despite its stronger *r*_p_ with happiness, family- and friendship-satisfaction. The *r*_g_ between wellbeing index and depressive symptoms or neuroticism (−0.67 or 0.55, respectively) were weaker than previously reported relationships with subjective wellbeing (−0.81 or −0.75, respectively) [8]. Factors influencing these estimates include different phenotype measurement, cohort size, and SNPs employed (e.g., the present study excluded the Major Histocompatibility Complex (MHC) region for *r*_g_ estimates). Furthermore, the genetic correlation between the wellbeing index and Okbay’s subjective wellbeing is 0.77, indicating the two scores are not identical; possibly due to inclusion of more elements of life satisfaction than affect in our score, which would diminish apparent relationships with neuroticism and depressive symptoms. One of the strengths of our study is using the same cohort and measures for different phenotypes, which makes genetic and phenotypic correlations more comparable – though we acknowledge that consistent reporting biases (e.g. relating to social desirability, or emotional state at the time of reporting) may influence associations derived from a single cohort.

The wellbeing index GWAS revealed five independent genome-wide significant loci; three consistent with previous reports [9, 10]. The chromosome 2 locus was previously associated with hedonic wellbeing [10]. Tagging SNP rs373377070 (max-*p* = 2.7 × 10^−9^) lies in an intron of *FSHR* (Follicle Stimulating Hormone Receptor), and influences its expression (GTeX v8; *p* = 1.1 × 10^−11^ [53]). Knockout mice (fshr^−/−^) display enhanced anxiety- and depression-like behaviours, and modulate gene expression in mood-mediating brain regions [54]. Two additional significant wellbeing-associated loci lie in the MHC/HLA region on chromosome 6, which is robustly associated with psychiatric conditions [30, 55]. One of these loci, tagged by rs3131073, was previously associated with positive affect and wellbeing spectrum [9, 10]. This suggests potentially pleiotropic effects of the MHC region on wellbeing and psychiatric illnesses. Two new loci identified on chromosomes 11 and 14 had minimal support from adjacent SNPs and may be spurious associations. Associations with related traits, including childhood maltreatment [16], are consistent with previously reported loci. Tissue-specific expression of mapped wellbeing-related genes using MAGMA showed significant enrichment of expression in brain tissues, consistent with previous reports [9]. Furthermore, the wellbeing index PGS significantly predicted the wellbeing index score and related phenotypes in the independent sample, albeit accounting for a small percentage of variance. Together, these data support the reliability of results reported herein, and the validity of the wellbeing index phenotype.

### Limitations and assumptions

One design limitation of our index score is that only subjective wellbeing indicators were included. The single item that related to psychological wellbeing was excluded due to the time-lag between baseline and follow-up assessments. Additionally, job satisfaction was excluded due to significant data missingness – representing individuals who were unemployed or retired at the time of assessment – which may have skewed the index score and limited generalisability, despite job satisfaction being an important component of wellbeing. Another caveat was the weak correlation (~0.25) of financial- and health-satisfaction to other items in the factor score. However, given their importance on subjective wellbeing [12, 56] and to maximise variable number to generate a quantitative index, we retained financial- and health-satisfaction as components in factor analysis. This led to moderate variance explained (47.5%) by the wellbeing index. Despite these limitations, the factor score had acceptable model fit in the confirmatory factor analysis and is a good proxy for subjective wellbeing.

Our study inherits the limitations of UK Biobank data. All the measures used in this study were self-report, with limited evidence of their validity, and reliability over time. The large sample sizes available through the UK Biobank enable highly significant associations to be derived from weak effect sizes, and caution should be taken in their interpretation. One should consider participant age (40–70 years), recall and reporting bias, and under-representation of psychiatric illnesses when interpreting results [43]. We cannot exclude misreports, longitudinal changes, and self-report bias [57] that could potentially influence our results. The confirmatory sample was ~6 years older than the discovery sample, and childhood maltreatment data came from follow-up assessments of participants who are better educated, with higher socioeconomic status and healthier than the baseline cohort and the general population [58]. Notably, our analyses were restricted to European-ancestry participants, which limits generalisability to other populations.

Finally, genetic correlations were estimated from common SNP variants identified via GWAS, and thus may underestimate pleiotropic contributions of other variant classes (e.g. rare SNVs or CNVs), and non-additive effects. Furthermore, as the SNP-heritability of the traits examined were small, only a small fraction of the causal SNPs for each phenotype contributed to the genetic correlations reported herein. While the LDSR method assumes that this small fraction is a random selection of all causal variants [22], recent studies have shown that these assumptions might not hold in similar complex traits to wellbeing such as human temperament [59]. LDSR also assumes that each phenotype is measured in ways that are specific, reliable and valid, and are well-matched across studies [23], which may not always be true and therefore may bias interpretation. Therefore, genetic correlations should be interpreted with caution, acknowledging the limitations and assumptions of the LDSR method.

### Conclusions and recommendations

Using a quantitative index of subjective wellbeing – which encompasses elements of general happiness and satisfaction with family, friendship, health, and financial situation – we explored the genetic and phenotypic relationships between positive and negative aspects of mental health and their correlation with mental illness and childhood maltreatment. The wellbeing index GWAS suggested that improving the measure by using multiple indicators of wellbeing could be a valid method to increase the explained genetic variance (SNP heritability) of wellbeing; although we note that unrealistic underlying models that are fundamental to PGS and LDSR that assume independent additive effects on traits in isolation from environmental exposures may contribute more substantially to ‘missing heritability’ and limit clinical utility. However, future studies will benefit from a combination of denser phenotyping, improved statistical genetic methods that are based on realistic assumptions (i.e. that reflect networks of genotype-phenotype effects across complex interrelated traits), and probably integration of technologies such as artificial intelligence and machine learning to advance the field. The negative association between childhood maltreatment and wellbeing, especially relating to emotional domains, highlights the importance of early life environment on wellbeing later in life. Most studies focus on the association of childhood maltreatment and psychopathologies; however, our study suggests that we might also need to measure the positive spectrum of mental health in relation to the childhood environment. Furthermore, our work may help to delineate the possible negative effects of maltreatment (i.e. on reduced wellbeing) before they become clinically significant. This is useful to know because indices of wellbeing can enable targeted provision of early support for people with an increased risk of developing psychiatric illnesses, which will segue into mental illness prevention. Finally, reduced wellbeing in the context of psychiatric illness, alongside a negative genetic correlation between wellbeing and psychiatric outcomes, demonstrates an interdependent relationship that can be influenced both by genetic and environmental factors, although directionality and causality remain to be determined in future studies. Determining the direction of effects and causality is possible using longitudinal studies with appropriate experimental design or genetic methods such as Mendelian randomisation. A better understanding of causality can then directly inform population-based interventions at societal or policy levels, to reduce the incidence of mental disorders and improve health and quality of life.

## Supplementary information

Supplementary Table S1

Supplementary Table S2

Supplementary Table S3

Supplementary Table S4

Supplementary Table S5

Supplementary Information

## Data Availability

The UKB Data described in the manuscript is available to all researchers and can be accessed upon approval of the UK Biobank (https://www.ukbiobank.ac.uk/enable-your-research/apply-for-access). We will return the derived data fields following UKB policy; in due course, they will be available through the UK Biobank Access Management System.

## Code availability

Analytic codes used in this study are stored in the UNSW data archive at www.dataarchive.unsw.edu.au, under Research Data Management Plan reference number H0237934, and are available on request.
