# Supplementary material for: Phenotypic and genetic analysis of a wellbeing factor score in the UK Biobank and the impact of childhood maltreatment and psychiatric illness
Source: Transl Psychiatry. 2022 Mar 19;12:113. doi: 10.1038/s41398-022-01874-5 (PMC8933416; doi:10.1038/s41398-022-01874-5)
Supplement: Supplementary file 1 — Supplementary Table S1 [file 41398_2022_1874_MOESM1_ESM.docx]

**Table S1. Demographic data for UK Biobank discovery cohort.** Females represented 53.84% of the discovery cohort, those reporting depressive symptoms represented 55.10%, those who had “Seen GP or Psychiatrist for nerves, anxiety, tension or depression” represented 34.84%, those reporting Loneliness/Isolation represented 17.26%, and 31.9% of the discovery cohort reported a college education (16.4% missing data).

| **Description** | **Data-Field ID** | **N** | **Mean ± SD** | **range** | **Variance** |
| --- | --- | --- | --- | --- | --- |
| Age at recruitment | 21022 | 129,237 | 57.276±8.009 | 40-70 | 64.151 |
| Happiness | 4526 | 129,237 | 4.463±0.695 | 1-6 | 0.483 |
| Friendship satisfaction | 4570 | 129,237 | 4.766±0.737 | 1-6 | 0.542 |
| Family satisfaction | 4559 | 129,237 | 4.799±0.892 | 1-6 | 0.795 |
| Financial satisfaction | 4581 | 129,237 | 4.315±0.937 | 1-6 | 0.878 |
| Health satisfaction | 4548 | 129,237 | 4.253±0.861 | 1-6 | 0.742 |
| Neuroticism | 20127 | 107,318 | 4.072±3.246 | 0-12 | 10.534 |
| Loneliness/Isolation | 2020 | 127,648 | 0.172±0.378 | 0-1 | 0.143 |
| Depressive symptoms | 4598 + 4631 | 122,720 | 0.551±0.497 | 0-1 | 0.247 |
| Seen GP or Psychiatrist | 2100 + 2090 | 128,221 | 0.348±0.476 | 0-1 | 0.227 |
| Wellbeing index score | N/A | 129,237 | 0.000±1.000 | -6.338-2.602 | 1 |
