# Supplementary material for: Phenotypic and genetic analysis of a wellbeing factor score in the UK Biobank and the impact of childhood maltreatment and psychiatric illness
Source: Transl Psychiatry. 2022 Mar 19;12:113. doi: 10.1038/s41398-022-01874-5 (PMC8933416; doi:10.1038/s41398-022-01874-5)
Supplement: Supplementary file 2 — Supplementary Table S2 [file 41398_2022_1874_MOESM2_ESM.docx]

**Table S2. Frequency of exposure to different types of childhood maltreatment and neglect in the sample (Data-Fields:20487-20490).**

*Abbreviations:* n, number; %, percentage of participants who answered the question.

*Notes:* Shaded categories are considered as trauma exposed. ^The physical neglect category (“...there was someone to take me to the doctor if I needed it”) was not included in the maltreatment sum score after analysis of the wellbeing index against the categorical responses indicating a potential misunderstanding of this question (Data-Field 20491). The people who answered “never” (n=893, mean=0.053) were more numerous than those who answered “rarely” (n=317) had a significantly higher wellbeing index score than both the population mean and the mean value of individual “exposed” subcategories (Supplementary Figure S3.e), suggesting that some participants may have responded “never” if they didn’t need to be taken to the doctor (i.e., they had no ailments), rather than not having someone to take them.

| **Response** | **Emotional neglect n (%)** | **Physical maltreatment n (%)** | **Emotional maltreatment n (%)** | **Sexual maltreatment n (%)** | **Physical neglect^^^ n (%)** |
| --- | --- | --- | --- | --- | --- |
| **Never true** | 583 (1.3%) | 37932 (81.8%) | 39360 (84.9%) | 42118 (91.6%) | 893 (1.9%) |
| **Rarely true** | 2097 (4.5%) | 4922 (10.6%) | 2736 (5.9%) | 2044 (4.4%) | 317 (0.7%) |
| **Sometimes true** | 7420 (16%) | 2899 (6.3%) | 2987 (6.4%) | 1392 (3%) | 1166 (2.5%) |
| **Often** | 11829 (25.5%) | 395 (0.9%) | 697 (1.5%) | 232 (0.5%) | 4831 (10.5%) |
| **Very often true** | 24377 (52.6%) | 226 (0.5%) | 564 (1.2%) | 176 (0.4%) | 38929 (84.4%) |
| **Total** | 46306 (100%) | 46374 (100%) | 46344 (100%) | 45962 (100%) | 46136 (100%) |
| **Prefer not to answer (missing)** | 151 | 83 | 113 | 495 | 321 |
