# Supplementary material for: Phenotypic and genetic analysis of a wellbeing factor score in the UK Biobank and the impact of childhood maltreatment and psychiatric illness
Source: Transl Psychiatry. 2022 Mar 19;12:113. doi: 10.1038/s41398-022-01874-5 (PMC8933416; doi:10.1038/s41398-022-01874-5)
Supplement: Supplementary file 3 — Supplementary Table S3 [file 41398_2022_1874_MOESM3_ESM.docx]

**Table S3. Genome-wide significant loci from GWAS of wellbeing index score.**

*Abbreviations: uniqID,* unique SNP identifier based on Human genome assembly GRCh37 (hg19) coordinates followed by allele 1 and allele 2; *rsID*, SNP identifier based on dbSNP; chr, chromosome; start, bp position of start of linkage disequilibrium block indexed by rsID; end, bp position of end of linkage disequilibrium block indexed by rsID; nSNPs, number of SNPs in LD block; nGWASSNPs, number of SNPs in LD block that were genome-wide significant; A1FREQ, frequency of allele 1; beta, effect size of SNP association; SE, standard error; P, p-value of SNP association with wellbeing index score.

*Acronyms:* SNP, single nucleotide polymorphism.

| **Genomic Locus** | **uniqID** | **rsID** | **chr** | **start** | **end** | **nSNPs** | **nGWAS**  **SNPs** | **A1FREQ** | **BETA** | **SE** | **P** |
| --- | --- | --- | --- | --- | --- | --- | --- | --- | --- | --- | --- |
| 1 | 2:49221769:G:GT | rs373377070 | 2 | 49211733 | 49240732 | 41 | 35 | 0.222 | 0.0279 | 0.0047 | 2.70E-09 |
| 2 | 6:28920972:A:G | rs3131073 | 6 | 27932301 | 29752808 | 437 | 369 | 0.880 | -0.0332 | 0.0060 | 3.00E-08 |
| 3 | 6:30223428:C:T | rs2189373 | 6 | 30122623 | 30312176 | 104 | 90 | 0.783 | -0.0285 | 0.0047 | 1.40E-09 |
| 4 | 11:127900579:A:G | rs1785039 | 11 | 127827727 | 127900579 | 2 | 2 | 0.818 | -0.0289 | 0.0050 | 8.80E-09 |
| 5 | 14:105887452:T:TGG | rs79167904 | 14 | 105883806 | 105916797 | 3 | 3 | 0.675 | -0.0245 | 0.0045 | 4.10E-08 |
