# Supplementary material for: Phenotypic and genetic analysis of a wellbeing factor score in the UK Biobank and the impact of childhood maltreatment and psychiatric illness
Source: Transl Psychiatry. 2022 Mar 19;12:113. doi: 10.1038/s41398-022-01874-5 (PMC8933416; doi:10.1038/s41398-022-01874-5)
Supplement: Supplementary file 4 — Supplementary Table S4 [file 41398_2022_1874_MOESM4_ESM.docx]

**Table S4. Genes that map positionally and functionally to the four genome-wide significant loci from GWAS of wellbeing index score using FUMA.**

*Abbreviations: ensg,* ensembl gene identifier; *chr*, chromosome; start, GRCh37/hg19 base pair position of gene start; end, GRCh37/hg19 base pair position of gene end; *pLI*, Exome Aggregation Consortium (ExAC) probability of being loss-of-function (LoF) intolerant, where pLI>0.9 indicates high likelihood of intolerance (refer to Lek *et al.* Nature 536:285-291, 2016); ncRVIS, residual variation intolerance score for non-coding variants (refer to Petrovski et al, PLoS Genet. 11(9):e1005492, 2015); posMapSNPs, The number of SNPs mapped to gene based on positional mapping; posMapCADD, The maximum CADD score of mapped SNPs by positional mapping; eqtlMapSNPs, The number of SNPs mapped to the gene based on eQTL mapping; eqtlMapminQ, The minimum eQTL FDR of mapped SNPs; eqtlDir., Consecutive direction of mapped eQTL SNPs after aligning risk increasing alleles in GWAS and tested alleles in eQTL data source; ciMap, "Yes" if the gene is mapped by chromatin interaction mapping; minGwasP, The minimum P-value of mapped SNPs; IndSigSNPs, rsID of the all independent significant SNPs of mapped SNPs; genomic locus, locus identifier from Table S3.

*Acronyms:* SNP, single nucleotide polymorphism; FDR, false discovery rate; CADD, Combined Annotation Dependent Depletion score (see Kircher et al, Nat Genet, 6(3):310-5, 2014)

| **ensg** | **gene**  **symbol** | **chr** | **start** | **end** | **pLI** | **ncRVIS** | **Pos**  **Map**  **SNPs** | **Pos**  **Map**  **CADD** | **eqtl**  **Map**  **SNPs** | **eqtl**  **Map**  **minQ** | **eqtl Dir.** | **ciMap** | **Min GwasP** | **IndSig­­SNPs** | **Genomic Locus** |
| --- | --- | --- | --- | --- | --- | --- | --- | --- | --- | --- | --- | --- | --- | --- | --- |
| ENSG00000170820 | FSHR | 2 | 49189296 | 49381676 | 3.75E-10 | 0.3335 | 41 | 19.16 | 0 | NA | NA | No | 2.70E-09 | rs373377070 | 1 |
| ENSG00000068781 | STON1-GTF2A1L | 2 | 48757064 | 49003654 | 1.65E-14 | 0.5729 | 0 | 0 | 1 | 5.65E-06 | + | No | 2.60E-08 | rs373377070 | 1 |
| **ENSG00000234127** | **TRIM26** | **6** | **30152232** | **30181204** | **0.829654** | **1.1226** | **34** | **14.54** | **117** | **7.55E-09** | **+** | **Yes** | **1.40E-09** | **rs2189373, rs3131073** | **2:3** |
| **ENSG00000235109** | **ZSCAN31** | **6** | **28292470** | **28324048** | **3.77E-09** | **1.1071** | **20** | **13.57** | **382** | **2.83E-21** | **+** | **Yes** | **3.00E-08** | **rs3131073** | **2** |
| ENSG00000204713 | TRIM27 | 6 | 28870779 | 28891766 | 0.878752 | -0.1421 | 15 | 18.69 | 0 | NA | NA | Yes | 1.30E-07 | rs3131073 | 2 |
| ENSG00000243729 | OR5V1 | 6 | 29323007 | 29399744 | 0.003443 | 0.3236 | 13 | 11.39 | 181 | 0.048673 | NA | No | 3.00E-08 | rs3131073 | 2 |
| **ENSG00000198315** | **ZKSCAN8** | **6** | **28109688** | **28127250** | **0.000232** | **-0.1425** | **12** | **13.42** | **332** | **9.79E-11** | **+** | **Yes** | **3.00E-08** | **rs3131073** | **2** |
| **ENSG00000137338** | **PGBD1** | **6** | **28249314** | **28270326** | **1.47E-05** | **-0.0801** | **11** | **14.75** | **218** | **9.81E-05** | **+** | **Yes** | **3.00E-08** | **rs3131073** | **2** |
| ENSG00000158691 | ZSCAN12 | 6 | 28346732 | 28367511 | 1.44E-05 | NA | 11 | 9.552 | 0 | NA | NA | Yes | 3.90E-06 | rs3131073 | 2 |
| ENSG00000204681 | GABBR1 | 6 | 29523406 | 29601753 | 0.99997 | 0.2389 | 10 | 14.02 | 0 | NA | NA | No | 3.40E-07 | rs3131073 | 2 |
| ENSG00000197935 | ZNF311 | 6 | 28962562 | 28973093 | 0.000129 | 0.3779 | 9 | 11.6 | 0 | NA | NA | Yes | 9.80E-08 | rs3131073 | 2 |
| **ENSG00000137185** | **ZSCAN9** | **6** | **28192664** | **28201260** | **4.10E-08** | **0.4612** | **9** | **7.251** | **337** | **3.39E-05** | **+** | **Yes** | **3.00E-08** | **rs3131073** | **2** |
| **ENSG00000197279** | **ZNF165** | **6** | **28048753** | **28057341** | **0.000563** | **0.2280** | **8** | **10.67** | **76** | **0.006875** | **-** | **Yes** | **1.10E-05** | **rs3131073** | **2** |
| **ENSG00000204709** | **C6orf100** | **6** | **28911654** | **28912314** | **NA** | **NA** | **8** | **3.641** | **3** | **0.006376** | **+** | **Yes** | **3.00E-08** | **rs3131073** | **2** |
| ENSG00000189298 | ZKSCAN3 | 6 | 28317691 | 28336947 | 0.000123 | -0.6524 | 7 | 13.57 | 48 | 2.08E-21 | - | Yes | 8.50E-07 | rs3131073 | 2 |
| ENSG00000204704 | OR2W1 | 6 | 29011990 | 29013017 | 0.086562 | 0.5315 | 7 | 6.576 | 0 | NA | NA | No | 1.80E-07 | rs3131073 | 2 |
| ENSG00000196812 | ZSCAN16 | 6 | 28092338 | 28097860 | 2.67E-06 | 0.1842 | 7 | 5.89 | 3 | 0.009 | - | No | 1.50E-05 | rs3131073 | 2 |
| ENSG00000213886 | UBD | 6 | 29523292 | 29527702 | 0.079896 | 0.4015 | 5 | 14.02 | 0 | NA | NA | No | 3.50E-07 | rs3131073 | 2 |
| ENSG00000112462 | OR12D3 | 6 | 29341200 | 29343068 | 1.34E-05 | 0.8835 | 5 | 11.39 | 0 | NA | NA | No | 4.80E-07 | rs3131073 | 2 |
| ENSG00000187626 | ZKSCAN4 | 6 | 28212401 | 28227011 | 6.74E-05 | 0.6616 | 5 | 10.52 | 0 | NA | NA | Yes | 2.30E-07 | rs3131073 | 2 |
| ENSG00000204695 | OR14J1 | 6 | 29274403 | 29275519 | 8.02E-08 | 1.3355 | 5 | 10.49 | 0 | NA | NA | No | 3.70E-07 | rs3131073 | 2 |
| ENSG00000204687 | MAS1L | 6 | 29454474 | 29455738 | 0.000466 | 0.6895 | 5 | 8.385 | 0 | NA | NA | No | 5.20E-07 | rs3131073 | 2 |
| ENSG00000204700 | OR2J2 | 6 | 29141311 | 29142351 | 0.010727 | 0.2713 | 4 | 5.147 | 0 | NA | NA | No | 1.90E-07 | rs3131073 | 2 |
| ENSG00000204694 | OR11A1 | 6 | 29393281 | 29424848 | 0.043484 | 1.2303 | 4 | 3.951 | 181 | 0.048673 | NA | No | 3.00E-08 | rs3131073 | 2 |
| ENSG00000204688 | OR2H1 | 6 | 29424958 | 29432105 | 0.044904 | -0.3583 | 4 | 3.951 | 0 | NA | NA | No | 7.60E-07 | rs3131073 | 2 |
| ENSG00000204657 | OR2H2 | 6 | 29555683 | 29556745 | 0.089946 | 0.6968 | 4 | 3.904 | 0 | NA | NA | No | 5.70E-07 | rs3131073 | 2 |
| ENSG00000204599 | TRIM39 | 6 | 30294256 | 30311506 | 0.999421 | -0.5776 | 3 | 12.77 | 0 | NA | NA | Yes | 6.90E-08 | rs2189373 | 3 |
| ENSG00000241370 | RPP21 | 6 | 30312908 | 30314661 | 0.082454 | 0.3235 | 3 | 12.77 | 0 | NA | NA | Yes | 6.90E-08 | rs2189373 | 3 |
| ENSG00000248167 | TRIM39-RPP21 | 6 | 30297359 | 30314631 | NA | NA | 3 | 12.77 | 0 | NA | NA | No | 6.90E-08 | rs2189373 | 3 |
| ENSG00000204610 | TRIM15 | 6 | 30130993 | 30140473 | 2.18E-11 | 0.0670 | 3 | 10.25 | 0 | NA | NA | No | 9.50E-08 | rs2189373 | 3 |
| ENSG00000204703 | OR2B3 | 6 | 29053985 | 29055090 | 0.001337 | 0.4588 | 3 | 9.377 | 0 | NA | NA | No | 1.90E-07 | rs3131073 | 2 |
| ENSG00000168787 | OR12D2 | 6 | 29364416 | 29365448 | 0.601033 | 0.4578 | 3 | 6.779 | 0 | NA | NA | No | 7.50E-07 | rs3131073 | 2 |
| **ENSG00000187987** | **ZSCAN23** | **6** | **28399707** | **28411279** | **0.002703** | **0.0707** | **3** | **2.102** | **390** | **1.07E-24** | **+** | **No** | **3.00E-08** | **rs3131073** | **2** |
| ENSG00000206474 | OR10C1 | 6 | 29407083 | 29408731 | 0.227592 | 0.3639 | 3 | 1.81 | 0 | NA | NA | No | 5.40E-07 | rs3131073 | 2 |
| ENSG00000232040 | SCAND3 | 6 | 28539407 | 28583989 | 9.71E-09 | -0.7850 | 2 | 10.48 | 9 | 0.006502 | + | No | 1.80E-06 | rs3131073 | 2 |
| ENSG00000204702 | OR2J1 | 6 | 29068386 | 29069658 | 0.017495 | NA | 2 | 9.793 | 0 | NA | NA | No | 1.80E-07 | rs3131073 | 2 |
| ENSG00000189134 | NKAPL | 6 | 28227098 | 28228736 | 0.000166 | 0.0513 | 2 | 5.18 | 0 | NA | NA | Yes | 2.30E-07 | rs3131073 | 2 |
| ENSG00000204613 | TRIM10 | 6 | 30119722 | 30128711 | 9.95E-06 | 3.2062 | 2 | 4.969 | 0 | NA | NA | No | 1.30E-07 | rs2189373 | 3 |
| ENSG00000204701 | OR2J3 | 6 | 29079668 | 29080603 | 0.028144 | 0.1129 | 2 | 1.351 | 0 | NA | NA | No | 2.30E-07 | rs3131073 | 2 |
| ENSG00000204614 | TRIM40 | 6 | 30103885 | 30116512 | 1.53E-07 | 1.1870 | 1 | 4.969 | 0 | NA | NA | No | 5.00E-06 | rs2189373 | 3 |
| ENSG00000198704 | GPX6 | 6 | 28471073 | 28495992 | 3.14E-05 | 0.7187 | 1 | 0.984 | 0 | NA | NA | No | 1.80E-06 | rs3131073 | 2 |
| ENSG00000124657 | OR2B6 | 6 | 27925019 | 27925960 | 0.040731 | -0.0982 | 1 | 0.002 | 0 | NA | NA | No | 1.40E-05 | rs3131073 | 2 |
| ENSG00000096654 | ZNF184 | 6 | 27418522 | 27440897 | 0.133749 | -0.5457 | 0 | 0 | 95 | 0.000601 | + | Yes | 1.00E-07 | rs3131073 | 2 |
| ENSG00000184348 | HIST1H2AK | 6 | 27805658 | 27806117 | 0.068429 | -0.0412 | 0 | 0 | 157 | 0.040686 | NA | Yes | 2.00E-07 | rs3131073 | 2 |
| ENSG00000197238 | HIST1H4J | 6 | 27791884 | 27792257 | 0.423754 | 0.5984 | 0 | 0 | 23 | 0.008753 | - | Yes | 2.30E-07 | rs3131073 | 2 |
| ENSG00000198558 | HIST1H4L | 6 | 27840926 | 27841289 | 0.191235 | 0.4269 | 0 | 0 | 25 | 0.019915 | - | Yes | 2.30E-07 | rs3131073 | 2 |
| ENSG00000079691 | LRRC16A | 6 | 25279306 | 25620758 | 0.502319 | 0.4007 | 0 | 0 | 0 | NA | NA | Yes | 1.50E-05 | rs3131073 | 2 |
| ENSG00000079689 | SCGN | 6 | 25652464 | 25702011 | 0.035946 | 0.1029 | 0 | 0 | 0 | NA | NA | Yes | 1.50E-05 | rs3131073 | 2 |
| ENSG00000124610 | HIST1H1A | 6 | 26017260 | 26018040 | 0.003531 | -0.3722 | 0 | 0 | 0 | NA | NA | Yes | 2.30E-07 | rs3131073 | 2 |
| ENSG00000198366 | HIST1H3A | 6 | 26020718 | 26021186 | 3.98E-05 | -0.2257 | 0 | 0 | 0 | NA | NA | Yes | 2.30E-07 | rs3131073 | 2 |
| ENSG00000196176 | HIST1H4A | 6 | 26021907 | 26022278 | 0.002285 | 0.4342 | 0 | 0 | 0 | NA | NA | Yes | 2.30E-07 | rs3131073 | 2 |
| ENSG00000124529 | HIST1H4B | 6 | 26027124 | 26027480 | 0.008542 | 0.1661 | 0 | 0 | 0 | NA | NA | Yes | 2.30E-07 | rs3131073 | 2 |
| ENSG00000124693 | HIST1H3B | 6 | 26031817 | 26032288 | 0.307283 | 0.2517 | 0 | 0 | 0 | NA | NA | Yes | 2.30E-07 | rs3131073 | 2 |
| ENSG00000137259 | HIST1H2AB | 6 | 26033320 | 26033796 | 0.005319 | 0.2281 | 0 | 0 | 0 | NA | NA | Yes | 2.30E-07 | rs3131073 | 2 |
| ENSG00000010704 | HFE | 6 | 26087509 | 26098571 | 8.50E-07 | 0.2102 | 0 | 0 | 0 | NA | NA | Yes | 2.30E-07 | rs3131073 | 2 |
| ENSG00000197061 | HIST1H4C | 6 | 26104104 | 26104518 | 8.86E-05 | 0.8522 | 0 | 0 | 0 | NA | NA | Yes | 2.30E-07 | rs3131073 | 2 |
| ENSG00000197409 | HIST1H3D | 6 | 26197068 | 26199521 | 0.011195 | NA | 0 | 0 | 0 | NA | NA | Yes | 1.50E-05 | rs3131073 | 2 |
| ENSG00000196866 | HIST1H2AD | 6 | 26199079 | 26199471 | 4.00E-05 | 0.2878 | 0 | 0 | 0 | NA | NA | Yes | 1.50E-05 | rs3131073 | 2 |
| ENSG00000197846 | HIST1H2BF | 6 | 26199748 | 26200942 | 0.0003 | 0.8511 | 0 | 0 | 0 | NA | NA | Yes | 1.50E-05 | rs3131073 | 2 |
| ENSG00000198518 | HIST1H4E | 6 | 26204858 | 26206266 | 0.000642 | 1.8358 | 0 | 0 | 0 | NA | NA | Yes | 1.50E-05 | rs3131073 | 2 |
| ENSG00000187990 | HIST1H2BG | 6 | 26216428 | 26216872 | 2.94E-06 | 0.0253 | 0 | 0 | 0 | NA | NA | Yes | 1.50E-05 | rs3131073 | 2 |
| ENSG00000168274 | HIST1H2AE | 6 | 26217165 | 26217711 | 0.624124 | -0.2739 | 0 | 0 | 0 | NA | NA | Yes | 1.50E-05 | rs3131073 | 2 |
| ENSG00000196966 | HIST1H3E | 6 | 26225383 | 26225844 | 0.000813 | -0.2098 | 0 | 0 | 0 | NA | NA | Yes | 1.50E-05 | rs3131073 | 2 |
| ENSG00000124575 | HIST1H1D | 6 | 26234440 | 26235216 | NA | 0.1361 | 0 | 0 | 0 | NA | NA | Yes | 1.50E-05 | rs3131073 | 2 |
| ENSG00000158406 | HIST1H4H | 6 | 26281283 | 26285762 | 0.00879 | 1.0591 | 0 | 0 | 0 | NA | NA | Yes | 1.50E-05 | rs3131073 | 2 |
| ENSG00000182952 | HMGN4 | 6 | 26538633 | 26546482 | 0.557082 | -0.3510 | 0 | 0 | 0 | NA | NA | Yes | 2.30E-07 | rs3131073 | 2 |
| ENSG00000146109 | ABT1 | 6 | 26597180 | 26600278 | 0.027106 | 0.1042 | 0 | 0 | 0 | NA | NA | Yes | 2.30E-07 | rs3131073 | 2 |
| ENSG00000124635 | HIST1H2BJ | 6 | 27093676 | 27100541 | 0.478842 | 0.3086 | 0 | 0 | 0 | NA | NA | Yes | 2.30E-07 | rs3131073 | 2 |
| ENSG00000196787 | HIST1H2AG | 6 | 27100832 | 27103070 | 0.00061 | -0.7416 | 0 | 0 | 0 | NA | NA | Yes | 2.30E-07 | rs3131073 | 2 |
| ENSG00000197903 | HIST1H2BK | 6 | 27106073 | 27114619 | 0.490754 | -0.0431 | 0 | 0 | 0 | NA | NA | Yes | 2.30E-07 | rs3131073 | 2 |
| ENSG00000198339 | HIST1H4I | 6 | 27107076 | 27108418 | 0.446296 | -0.4680 | 0 | 0 | 0 | NA | NA | Yes | 2.30E-07 | rs3131073 | 2 |
| ENSG00000184825 | HIST1H2AH | 6 | 27114861 | 27115317 | 6.30E-05 | 0.1949 | 0 | 0 | 0 | NA | NA | Yes | 2.30E-07 | rs3131073 | 2 |
| ENSG00000158553 | POM121L2 | 6 | 27253682 | 27279949 | NA | 0.3134 | 0 | 0 | 0 | NA | NA | Yes | 2.30E-07 | rs3131073 | 2 |
| ENSG00000185130 | HIST1H2BL | 6 | 27775257 | 27775709 | 0.002868 | -0.0301 | 0 | 0 | 0 | NA | NA | Yes | 2.30E-07 | rs3131073 | 2 |
| ENSG00000196747 | HIST1H2AI | 6 | 27775899 | 27776429 | 0.581587 | -0.0583 | 0 | 0 | 0 | NA | NA | Yes | 2.30E-07 | rs3131073 | 2 |
| ENSG00000203813 | HIST1H3H | 6 | 27777842 | 27778314 | 0.024939 | -0.1778 | 0 | 0 | 0 | NA | NA | Yes | 2.30E-07 | rs3131073 | 2 |
| ENSG00000182611 | HIST1H2AJ | 6 | 27782112 | 27782607 | 0.191198 | 0.6151 | 0 | 0 | 0 | NA | NA | Yes | 2.30E-07 | rs3131073 | 2 |
| ENSG00000196374 | HIST1H2BM | 6 | 27782822 | 27783267 | 0.002753 | -0.2756 | 0 | 0 | 0 | NA | NA | Yes | 2.30E-07 | rs3131073 | 2 |
| ENSG00000197914 | HIST1H4K | 6 | 27798952 | 27799305 | 0.429119 | 0.3874 | 0 | 0 | 0 | NA | NA | Yes | 2.30E-07 | rs3131073 | 2 |
| ENSG00000233822 | HIST1H2BN | 6 | 27806323 | 27823487 | 0.139775 | 0.1455 | 0 | 0 | 0 | NA | NA | Yes | 2.30E-07 | rs3131073 | 2 |
| ENSG00000198374 | HIST1H2AL | 6 | 27833034 | 27833606 | 0.062487 | -0.3603 | 0 | 0 | 0 | NA | NA | Yes | 2.30E-07 | rs3131073 | 2 |
| ENSG00000184357 | HIST1H1B | 6 | 27834570 | 27835359 | 0.509139 | -0.0899 | 0 | 0 | 0 | NA | NA | Yes | 2.30E-07 | rs3131073 | 2 |
| ENSG00000182572 | HIST1H3I | 6 | 27839623 | 27840099 | 0.36088 | -0.2324 | 0 | 0 | 0 | NA | NA | Yes | 2.30E-07 | rs3131073 | 2 |
| ENSG00000197153 | HIST1H3J | 6 | 27858093 | 27860884 | 0.695884 | -0.1897 | 0 | 0 | 0 | NA | NA | Yes | 1.00E-07 | rs3131073 | 2 |
| ENSG00000233224 | HIST1H2AM | 6 | 27860477 | 27860963 | 0.000856 | -0.2251 | 0 | 0 | 0 | NA | NA | Yes | 1.00E-07 | rs3131073 | 2 |
| ENSG00000196331 | HIST1H2BO | 6 | 27861203 | 27861669 | 3.07E-05 | -0.2231 | 0 | 0 | 0 | NA | NA | Yes | 1.00E-07 | rs3131073 | 2 |
| ENSG00000204525 | HLA-C | 6 | 31236526 | 31239907 | 9.95E-10 | 8.4419 | 0 | 0 | 447 | 3.69E-23 | + | No | 3.00E-08 | rs2189373; rs3131073 | 2:3 |
| ENSG00000137312 | FLOT1 | 6 | 30695486 | 30710510 | 0.015273 | NA | 0 | 0 | 83 | 4.53E-11 | + | No | 3.30E-08 | rs2189373; rs3131073 | 2:3 |
| ENSG00000112812 | PRSS16 | 6 | 27215480 | 27224403 | 2.24E-06 | 0.0088 | 0 | 0 | 73 | 3.97E-11 | + | No | 1.10E-05 | rs3131073 | 2 |
| ENSG00000137411 | VARS2 | 6 | 30876019 | 30894236 | 4.22E-14 | NA | 0 | 0 | 303 | 8.33E-22 | - | No | 1.40E-09 | rs2189373; rs3131073 | 2:3 |
| ENSG00000204540 | PSORS1C1 | 6 | 31082527 | 31107869 | 0.000855 | 2.3727 | 0 | 0 | 76 | 3.14E-11 | - | No | 3.30E-08 | rs2189373 | 3 |
| ENSG00000206503 | HLA-A | 6 | 29909037 | 29913661 | 0.13278 | 11.8053 | 0 | 0 | 23 | 1.42E-07 | - | No | 1.40E-09 | rs2189373 | 3 |
| ENSG00000204632 | HLA-G | 6 | 29794744 | 29798902 | 9.55E-08 | 1.6580 | 0 | 0 | 234 | 1.04E-09 | - | No | 3.00E-08 | rs3131073;  rs2189373 | 2:3 |
| ENSG00000204642 | HLA-F | 6 | 29690552 | 29706305 | 4.40E-05 | 0.7343 | 0 | 0 | 343 | 1.45E-17 | - | No | 1.40E-09 | rs3131073;  rs2189373 | 2:3 |
| ENSG00000204536 | CCHCR1 | 6 | 31110216 | 31126015 | 6.50E-08 | NA | 0 | 0 | 78 | 1.32E-10 | + | No | 1.40E-09 | rs2189373 | 3 |
| ENSG00000244731 | C4A | 6 | 31949801 | 31970458 | NA | -0.3186 | 0 | 0 | 483 | 0.009 | - | No | 1.40E-09 | rs3131073;  rs2189373 | 2:3 |
| ENSG00000111801 | BTN3A3 | 6 | 26440700 | 26453643 | 7.19E-10 | 1.0901 | 0 | 0 | 359 | 0.009 | + | No | 3.00E-08 | rs3131073 | 2 |
| ENSG00000204592 | HLA-E | 6 | 30457244 | 30461982 | 0.347084 | -0.0809 | 0 | 0 | 90 | 0.009 | + | No | 3.30E-08 | rs3131073;  rs2189373 | 2:3 |
| ENSG00000186470 | BTN3A2 | 6 | 26365387 | 26378546 | 0.000125 | 2.2079 | 0 | 0 | 42 | 0.009 | - | No | 1.10E-05 | rs3131073 | 2 |
| ENSG00000213780 | GTF2H4 | 6 | 30875961 | 30881883 | 0.258035 | 0.3444 | 0 | 0 | 19 | 0.049 | - | No | 3.30E-07 | rs2189373 | 3 |
| ENSG00000196230 | TUBB | 6 | 30687978 | 30693203 | 0.875277 | 0.9715 | 0 | 0 | 2 | 0.049 | - | No | 1.40E-07 | rs2189373 | 3 |
| ENSG00000226174 | TEX22 | 14 | 105864916 | 105916443 | NA | -0.1547 | 3 | 7.226 | 0 | NA | NA | No | 4.10E-08 | rs79167904 | 5 |
| ENSG00000182979 | MTA1 | 14 | 105886159 | 105937066 | 0.999748 | NA | 3 | 7.226 | 0 | NA | NA | No | 4.10E-08 | rs79167904 | 5 |
| ENSG00000166428 | PLD4 | 14 | 105391153 | 105399574 | 1.23E-13 | NA | 0 | 0 | 0 | NA | NA | Yes | 4.10E-08 | rs79167904 | 5 |
| ENSG00000140104 | C14orf79 | 14 | 105452112 | 105476819 | 5.13E-08 | -0.6203 | 0 | 0 | 0 | NA | NA | Yes | 4.10E-08 | rs79167904 | 5 |
| ENSG00000184986 | TMEM121 | 14 | 105992940 | 105996539 | 0.178807 | NA | 0 | 0 | 0 | NA | NA | Yes | 4.10E-08 | rs79167904 | 5 |
