# Supplementary material for: Phenotypic and genetic analysis of a wellbeing factor score in the UK Biobank and the impact of childhood maltreatment and psychiatric illness
Source: Transl Psychiatry. 2022 Mar 19;12:113. doi: 10.1038/s41398-022-01874-5 (PMC8933416; doi:10.1038/s41398-022-01874-5)
Supplement: Supplementary file 5 — Supplementary Table S5 [file 41398_2022_1874_MOESM5_ESM.docx]

**Table S5. FUMA query of GWAS catalog for SNPs significantly associated with wellbeing index to identify signal overlaps amongst related traits.**

*Abbreviations: Genomic locus*, Index of genomic risk loci; *IndSigSNP*, One of the independent significant SNPs of the SNP in GWAScatalog; *chr*, chromosome; *snp*, rsID of reported SNP in GWAS catalog; *PMID*, PubMed ID; *Trait*, The trait reported in GWAScatalog; *ReportedGene*, Gene(s) reported by author; *MappedGene*, Gene(s) mapped to the strongest SNP; *Strongest,* SNP(s) most strongly associated with trait + risk allele (? for unknown risk allele); *Variant* *Context,* SNP functional class; *RiskAF,* Reported risk/effect allele frequency associated with strongest SNP in controls; *P,* Reported p-value for strongest SNP risk allele (linked to dbGaP Association Browser); *Pmlog*, -log(p-value); *OrBeta,* Reported odds ratio or beta-coefficient associated with strongest SNP risk allele.
Acronyms: SNP, single nucleotide polymorphism; GWAS, genome wide association study.

| **Genomic Locus** | **IndSigSNP** | **snp** | **PMID** | **FirstAuthor** | **Trait** | **Reported Gene** | **Mapped Gene** | **Strongest** | **Variant Context** | **RiskAF** | **P** | **Pmlog** | **OrBeta** |
| --- | --- | --- | --- | --- | --- | --- | --- | --- | --- | --- | --- | --- | --- |
| **1** | **rs373377070** | **rs6732220** | **30279531** | **Baselmans** | **Hedonic well-being** | **NR** | **AC009975.1, FSHR** | **rs6732220-C** | **intron** | **0.770** | **4.0E-08** | **7.40** | **0.020** |
| 2 | rs3131073 | rs149949 | 29500382 | Nagel | Feeling guilty | NR | OR2W2P - OR2B7P | rs149949-T | intergenic | 0.146 | 3.0E-08 | 7.52 | 5.540 |
| 2 | rs3131073 | rs202906 | 28540026 | Anney | Autism spectrum disorder or schizophrenia | many | OR2W2P - OR2B7P | rs202906-T | intergenic | - | 3.0E-16 | 15.52 | 1.130 |
| 2 | rs3131073 | rs13197574 | 29662059 | Howard | Depression (broad) | NR | ZSCAN12P1 | rs13197574-C | nc_exon | 0.110 | 1.0E-11 | 11.00 | 0.013 |
| 2 | rs3131073 | rs66886492 | 29844566 | Davies | General cognitive ability | ZSCAN16-AS1 | ZSCAN16-AS1 | rs66886492-T | nc_exon | NR | 2.0E-15 | 14.70 | 7.943 |
| 2 | rs3131073 | rs66886492 | 30643256 | Baselmans | Depressive symptoms | NR | ZSCAN16-AS1 | rs66886492-T | nc_exon | 0.101 | 3.0E-15 | 14.52 | 0.017 |
| 2 | rs3131073 | rs9380069 | 29844566 | Davies | General cognitive ability | NR | ZSCAN9 - ZKSCAN4 | rs9380069-? | regulatory | NR | 7.0E-08 | 7.15 | 5.392 |
| 2 | rs3131073 | rs17720293 | 30285260 | Ikeda | Schizophrenia | ZKSCAN4 | ZKSCAN4 | rs17720293-C | intron | NR | 3.0E-23 | 22.52 | 1.197 |
| 2 | rs3131073 | rs853679 | 27089181 | Okbay | Depression | NR | ZSCAN31 | rs853679-A | intron | NR | 1.0E-07 | 7.00 | 5.298 |
| 2 | rs3131073 | rs13217619 | 26198764 | Goes | Schizophrenia | NR | ZSCAN31 | rs13217619-T | intron | NR | 2.0E-29 | 28.70 | 1.250 |
| **2** | **rs3131073** | **rs35016036** | **30643256** | **Baselmans** | **Positive affect** | **NR** | **ZSCAN31** | **rs35016036-T** | **intron** | **0.101** | **8.0E-09** | **8.10** | **0.021** |
| 2 | rs3131073 | rs35017208 | 29662059 | Howard | Depression (broad) | NR | ZKSCAN3 - ZSCAN12 | rs35017208-T | intergenic | 0.111 | 1.0E-12 | 12.00 | 0.013 |
| 2 | rs3131073 | rs13213152 | 30643256 | Baselmans | Depressive symptoms | NR | ZSCAN12 | rs13213152-G | 3'UTR | 0.102 | 7.0E-21 | 20.15 | 0.018 |
| **2** | **rs3131073** | **rs13213152** | **30643256** | **Baselmans** | **Well-being spectrum (multivariate analysis)** | **NR** | **ZSCAN12** | **rs13213152-G** | **3'UTR** | **0.102** | **6.0E-21** | **20.22** | **0.014** |
| 2 | rs3131073 | rs13191038 | 29662059 | Howard | Depression (broad) | NR | GPX6, GPX6 | rs13191038-C | intron | 0.110 | 4.0E-12 | 11.40 | 0.013 |
| 2 | rs3131073 | rs13194504 | 29662059 | Howard | Depression (broad) | NR | LINC00533 - RPSAP2 | rs13194504-A | intergenic | 0.112 | 1.0E-11 | 11.00 | 0.013 |
| 2 | rs3131073 | rs116137698 | 28540026 | Anney | Autism spectrum disorder or schizophrenia | many | LINC00533 - RPSAP2 | rs116137698-A | intergenic | - | 3.0E-26 | 25.52 | 1.220 |
| 2 | rs3131073 | rs7775835 | 29844566 | Davies | General cognitive ability | RPSAP2 | LINC00533 - RPSAP2 | rs7775835-T | intergenic | NR | 9.0E-12 | 11.05 | 6.824 |
| 2 | rs3131073 | rs7775835 | 29942085 | Nagel | Depression | NR | LINC00533 - RPSAP2 | rs7775835-T | intergenic | NR | 2.0E-08 | 7.70 | 5.588 |
| 2 | rs3131073 | rs115329265 | 25056061 | Ripke | Schizophrenia | intergenic | RPSAP2 - NOP56P1 | rs115329265-A | intergenic | 0.850 | 3.0E-31 | 30.52 | 1.205 |
| 2 | rs3131073 | rs115329265 | 28540026 | Anney | Autism spectrum disorder or schizophrenia | many | RPSAP2 - NOP56P1 | rs115329265-A | intergenic | - | 2.0E-27 | 26.70 | 1.170 |
| 2 | rs3131073 | rs115329265 | 28991256 | Li | Schizophrenia | ZKSCAN3, ZNF323, ZSCAN23 | RPSAP2 - NOP56P1 | rs115329265-A | intergenic | NR | 5.0E-36 | 35.30 | 1.210 |
| 2 | rs3131073 | rs115329265 | 30285260 | Ikeda | Schizophrenia | Intergenic | RPSAP2 - NOP56P1 | rs115329265-A | intergenic | NR | 6.0E-33 | 32.22 | 1.217 |
| 2 | rs3131073 | rs1233578 | 29942086 | Savage | Intelligence | NR | RPSAP2 - NOP56P1 | rs1233578-A | intergenic | NR | 1.0E-09 | 9.00 | 6.098 |
| 2 | rs3131073 | rs1233578 | 29844566 | Davies | General cognitive ability | RPSAP2 | RPSAP2 - NOP56P1 | rs1233578-A | intergenic | NR | 5.0E-12 | 11.30 | 6.899 |
| 2 | rs3131073 | rs9257248 | 29662059 | Howard | Depression (broad) | NR | NOP56P1 - AL662890.1 | rs9257248-T | intergenic | 0.114 | 4.0E-11 | 10.40 | 0.012 |
| 2 | rs3131073 | rs3129791 | 29662059 | Howard | Depression (broad) | NR | HCG15 | rs3129791-A | intron | 0.115 | 2.0E-11 | 10.70 | 0.013 |
| 2 | rs3131073 | rs116757206 | 30285260 | Ikeda | Schizophrenia | ZNF311 | ZNF311 - OR2AD1P | rs116757206-G | regulatory | NR | 1.0E-25 | 25.00 | 1.239 |
| 2 | rs3131073 | rs4947263 | 29662059 | Howard | Depression (broad) | NR | AL645937.4 | rs4947263-T | nc_exon | 0.118 | 3.0E-12 | 11.52 | 0.013 |
| 2 | rs3131073 | rs150817755 | 30285260 | Ikeda | Schizophrenia | OR2G1P | AL672167.1 | rs150817755-T | intron | NR | 1.0E-29 | 29.00 | 1.261 |
| 2 | rs3131073 | rs3130820 | 29483656 | Pardinas | Schizophrenia | xMHC | AL672167.1 | rs3130820-? | intron | NR | 2.0E-44 | 43.70 | 1.281 |
| 2 | rs3131073 | rs144447022 | 26198764 | Goes | Schizophrenia | NR | AL672167.1 | rs144447022-G | intron | NR | 2.0E-28 | 27.70 | 1.250 |
| 2 | rs3131073 | rs144447022 | 29326435 | Hill | Intelligence (MTAG) | XXbac-BPG308J9.3 | AL672167.1 | rs144447022-T | intron | NR | 1.0E-13 | 13.00 | 0.036 |
| 2 | rs3131073 | rs9257681 | 29662059 | Howard | Depression (broad) | NR | AL672167.1 | rs9257681-G | intron | 0.115 | 2.0E-12 | 11.70 | 0.013 |
| 2 | rs3131073 | rs3117425 | 29942085 | Nagel | Worry | NR | AL672167.1 | rs3117425-T | nc_exon | NR | 2.0E-08 | 7.70 | 0.020 |
| 2 | rs3131073 | rs3117425 | 29500382 | Nagel | Feeling nervous | ZNF391 | AL672167.1 | rs3117425-T | nc_exon | 0.119 | 5.0E-08 | 7.30 | 5.470 |
| 2 | rs3131073 | rs114071887 | 30285260 | Ikeda | Schizophrenia | OR12D3 | OR5V1, OR12D3 | rs114071887-G | missense | NR | 7.0E-25 | 24.15 | 1.239 |
| 2 | rs3131073 | rs3749971 | 29942086 | Savage | Intelligence | NR | OR5V1, OR12D3 | rs3749971-A | missense | NR | 3.0E-09 | 8.52 | 5.929 |
| 2 | rs3131073 | rs429479 | 29844566 | Davies | General cognitive ability | OR5V1 | OR5V1 | rs429479-A | intron | NR | 5.0E-15 | 14.30 | 7.818 |
| 2 | rs3131073 | rs2523443 | 29662059 | Howard | Depression (broad) | NR | OR11A1 | rs2523443-A | intron | 0.119 | 1.0E-12 | 12.00 | 0.013 |
| 2 | rs3131073 | rs1233393 | 29662059 | Howard | Depression (broad) | NR | GABBR1 | rs1233393-G | intron | 0.122 | 8.0E-13 | 12.10 | 0.013 |
| 2 | rs3131073 | rs9258375 | 29662059 | Howard | Depression (broad) | NR | AL645939.3 - HLA-V | rs9258375-G | regulatory | 0.122 | 1.0E-12 | 12.00 | 0.013 |
| 2 | rs3131073 | rs149943 | 29899525 | Klimentidis | Moderate to vigorous physical activity levels | ZNF165 | OR2W2P | rs149943-G | nc_exon | 0.850 | 2.0E-09 | 8.70 | 0.019 |
| 2 | rs3131073 | rs149949 | 28604730 | McKay | Squamous cell lung carcinoma | OR2B6, ZNF165 | OR2W2P - OR2B7P | rs149949-C | intergenic | 0.102 | 2.0E-10 | 9.70 | 1.206 |
| 2 | rs3131073 | rs13197574 | 26651848 | Rivera | Sarcoidosis (Lofgren's syndrome vs non-Lofgren's syndrome) | ZNF165, LOC100129195 | ZSCAN12P1 | rs13197574-G | nc_exon | 0.208 | 3.0E-17 | 16.52 | - |
| 2 | rs3131073 | rs34662244 | 28604730 | McKay | Lung cancer in ever smokers | ZSCAN12P1, ZSCAN16-AS1 | ZSCAN12P1 - ZNF602P | rs34662244-A | intergenic | 0.086 | 2.0E-09 | 8.70 | 1.176 |
| 2 | rs3131073 | rs34662244 | 29059683 | Michailidou | Breast cancer | NR | ZSCAN12P1 - ZNF602P | rs34662244-A | intergenic | 0.092 | 3.0E-09 | 8.52 | 0.069 |
| 2 | rs3131073 | rs34662244 | 28928442 | Tian | Urinary tract infection frequency | NR | ZSCAN12P1 - ZNF602P | rs34662244-? | intergenic | NR | 1.0E-06 | 6.00 | 0.060 |
| 2 | rs3131073 | rs35952432 | 28604730 | McKay | Lung cancer | ZSCAN12P1, ZSCAN16-AS1 | ZSCAN12P1 - ZNF602P | rs35952432-T | intergenic | 0.085 | 6.0E-11 | 10.22 | 1.148 |
| 2 | rs3131073 | rs17720293 | 28928442 | Tian | Urinary tract infection frequency | NR | ZKSCAN4 | rs17720293-? | intron | NR | 1.0E-06 | 6.00 | 0.057 |
| 2 | rs3131073 | rs34878803 | 28604730 | McKay | Squamous cell lung carcinoma | PGBD1 | PGBD1 | rs34878803-C | intron | 0.077 | 4.0E-11 | 10.40 | 1.251 |
| 2 | rs3131073 | rs33932084 | 28240269 | Suhre | Blood protein levels | many | PGBD1 | rs33932084-G | missense | 0.075 | 4.0E-16 | 15.40 | 0.672 |
| 2 | rs3131073 | rs33932084 | 30578418 | Giri | Pulse pressure | PGBD1 | PGBD1 | rs33932084-G | missense | 0.909 | 6.0E-15 | 14.22 | 0.340 |
| 2 | rs3131073 | rs34661125 | 28604730 | McKay | Lung cancer | PGBD1, ZSCAN31 | PGBD1 - AL021997.2 | rs34661125-A | intergenic | 0.079 | 2.0E-12 | 11.70 | 1.160 |
| 2 | rs3131073 | rs7752448 | 30804560 | Shrine | FEV1 | ZNF184 | ZSCAN31 | rs7752448-A | intron | 0.875 | 3.0E-35 | 34.52 | 0.044 |
| 2 | rs3131073 | rs7752448 | 30804560 | Shrine | Lung function (FEV1/FVC) | ZNF184 | ZSCAN31 | rs7752448-A | intron | 0.875 | 7.0E-16 | 15.15 | 0.029 |
| 2 | rs3131073 | rs7752448 | 30804560 | Shrine | Lung function (FVC) | ZNF184 | ZSCAN31 | rs7752448-A | intron | 0.875 | 1.0E-22 | 22.00 | 0.035 |
| 2 | rs3131073 | rs7752448 | 30804560 | Shrine | Peak expiratory flow | ZNF184 | ZSCAN31 | rs7752448-A | intron | 0.875 | 1.0E-48 | 48.00 | 0.055 |
| 2 | rs3131073 | rs67340775 | 28604730 | McKay | Lung cancer in ever smokers | ZSCAN31 | ZSCAN31 | rs67340775-C | intron | 0.079 | 7.0E-10 | 9.15 | 1.182 |
| 2 | rs3131073 | rs13214023 | 29059683 | Michailidou | Breast cancer | NR | ZKSCAN3 | rs13214023-A | intron | 0.093 | 1.0E-09 | 9.00 | 0.071 |
| 2 | rs3131073 | rs13213152 | 26651848 | Rivera | Sarcoidosis (Lofgren's syndrome vs non-Lofgren's syndrome) | ZKSCAN3, ZSCAN12 | ZSCAN12 | rs13213152-G | 3'UTR | 0.212 | 5.0E-18 | 17.30 | - |
| 2 | rs3131073 | rs13213986 | 28604730 | McKay | Squamous cell lung carcinoma | ZSCAN12 | ZSCAN12 | rs13213986-A | intron | 0.077 | 7.0E-11 | 10.15 | 1.249 |
| 2 | rs3131073 | rs13201681 | 28604730 | McKay | Lung cancer | ZSCAN12, ZSCAN23 | RNU2-45P - ZSCAN23 | rs13201681-T | intergenic | 0.078 | 7.0E-12 | 11.15 | 1.157 |
| 2 | rs3131073 | rs67381177 | 28604730 | McKay | Lung cancer in ever smokers | ZSCAN23 | ZSCAN23 - COX11P1 | rs67381177-C | regulatory | 0.079 | 2.0E-09 | 8.70 | 1.178 |
| 2 | rs3131073 | rs114242735 | 28604730 | McKay | Squamous cell lung carcinoma | GPX6 | GPX6, GPX6 | rs114242735-C | intron | 0.077 | 2.0E-10 | 9.70 | 1.242 |
| 2 | rs3131073 | rs13191038 | 28928442 | Tian | Urinary tract infection frequency | NR | GPX6, GPX6 | rs13191038-? | intron | NR | 5.0E-06 | 5.30 | 0.058 |
| 2 | rs3131073 | rs114385935 | 28604730 | McKay | Lung cancer in ever smokers | ZBED9, LINC01623 | LINC00533 - RPSAP2 | rs114385935-G | intergenic | 0.080 | 7.0E-09 | 8.15 | 1.170 |
| 2 | rs3131073 | rs6908726 | 30643258 | Karlsson | Number of sexual partners | ZBED9 | LINC00533 - RPSAP2 | rs6908726-C | intergenic | 0.875 | 2.0E-13 | 12.70 | 0.026 |
| 2 | rs3131073 | rs1233578 | 29273806 | Demenais | Asthma | GPX5, TRIM27 | RPSAP2 - NOP56P1 | rs1233578-G | intergenic | 0.130 | 6.0E-07 | 6.22 | 1.090 |
| 2 | rs3131073 | rs1233578 | 29273806 | Demenais | Asthma | GPX5, TRIM27 | RPSAP2 - NOP56P1 | rs1233578-G | intergenic | 0.130 | 5.0E-09 | 8.30 | 0.100 |
| 2 | rs3131073 | rs4324798 | 19836008 | Landi | Lung adenocarcinoma | TRNAA-UGC | NOP56P1 - AL662890.1 | rs4324798-A | intergenic | 0.090 | 2.0E-08 | 7.70 | 1.160 |
| 2 | rs3131073 | rs3118359 | 30275531 | Klarin | Triglycerides | intergenic | NOP56P1 - AL662890.1 | rs3118359-T | intergenic | 0.084 | 3.0E-13 | 12.52 | 0.038 |
| 2 | rs3131073 | rs116461399 | 28604730 | McKay | Lung cancer | ZBED9, LINC01623 | NOP56P1 - AL662890.1 | rs116461399-C | regulatory | 0.096 | 1.0E-12 | 12.00 | 1.155 |
| 2 | rs3131073 | rs116461399 | 28604730 | McKay | Squamous cell lung carcinoma | ZBED9, LINC01623 | NOP56P1 - AL662890.1 | rs116461399-C | regulatory | 0.097 | 1.0E-11 | 11.00 | 1.247 |
| 2 | rs3131073 | rs3131337 | 28928442 | Tian | Cold sores | NR | NOP56P1 - AL662890.1 | rs3131337-? | regulatory | NR | 7.0E-06 | 5.15 | 0.081 |
| 2 | rs3131073 | rs9257248 | 26651848 | Rivera | Sarcoidosis (Lofgren's syndrome vs non-Lofgren's syndrome) | LOC442181, LOC401242 | NOP56P1 - AL662890.1 | rs9257248-A | intergenic | 0.217 | 7.0E-20 | 19.15 | - |
| 2 | rs3131073 | rs116381494 | 28604730 | McKay | Lung cancer in ever smokers | HCG14, TRIM27 | HCG14 - TRIM27 | rs116381494-G | regulatory | 0.083 | 6.0E-08 | 7.22 | 1.168 |
| 2 | rs3131073 | rs3130895 | 26651848 | Rivera | Sarcoidosis (Lofgren's syndrome vs non-Lofgren's syndrome) | TRIM27, C6orf100 | TRIM27 - LINC01556 | rs3130895-A | intergenic | 0.218 | 4.0E-19 | 18.40 | - |
| 2 | rs3131073 | rs3130844 | 28928442 | Tian | Urinary tract infection frequency | NR | LINC01556 - KRT18P1 | rs3130844-? | regulatory | NR | 7.0E-06 | 5.15 | 0.056 |
| 2 | rs3131073 | rs148696809 | 28604730 | McKay | Lung cancer | LINC01556, ZNF311 | LINC01556 - KRT18P1 | rs148696809-C | intergenic | 0.087 | 3.0E-12 | 11.52 | 1.157 |
| 2 | rs3131073 | rs148696809 | 28604730 | McKay | Squamous cell lung carcinoma | LINC01556, ZNF311 | LINC01556 - KRT18P1 | rs148696809-C | intergenic | 0.087 | 4.0E-11 | 10.40 | 1.248 |
| 2 | rs3131073 | rs148696809 | 30038396 | Lee | Cognitive performance | Intergenic | LINC01556 - KRT18P1 | rs148696809-T | intergenic | 0.883 | 8.0E-20 | 19.10 | 0.041 |
| 2 | rs3131073 | rs115287935 | 28604730 | McKay | Lung cancer in ever smokers | ZNF311, LOC100129636 | ZNF311 - OR2AD1P | rs115287935-T | intergenic | 0.082 | 9.0E-08 | 7.05 | 1.165 |
| 2 | rs3131073 | rs3131082 | 28928442 | Tian | Tuberculosis | NR | ZNF311 - OR2AD1P | rs3131082-? | regulatory | NR | 2.0E-07 | 6.70 | 0.195 |
| 2 | rs3131073 | rs3131085 | 28240269 | Suhre | Blood protein levels | many | SAR1AP1 | rs3131085-A | nc_exon | 0.076 | 1.0E-09 | 9.00 | 0.511 |
| 2 | rs3131073 | rs3129788 | 26651848 | Rivera | Sarcoidosis (Lofgren's syndrome vs non-Lofgren's syndrome) | OR2B3P, OR2J3 | OR2B3 - OR2J1 | rs3129788-A | regulatory | 0.217 | 7.0E-19 | 18.15 | - |
| 2 | rs3131073 | rs3129178 | 29059683 | Michailidou | Breast cancer | NR | OR2J1 - OR2J3 | rs3129178-A | intergenic | 0.095 | 6.0E-09 | 8.22 | 0.067 |
| 2 | rs3131073 | rs116442863 | 28604730 | McKay | Lung cancer in ever smokers | OR2J3, OR2J2 | OR2J3 - AL645937.2 | rs116442863-A | intergenic | 0.082 | 1.0E-07 | 7.00 | 1.163 |
| 2 | rs3131073 | rs3130725 | 27668658 | Iotchkova | Cardiometabolic and hematological traits | ZNF311 | AL645937.1 - AL645937.4 | rs3130725-G | intergenic | - | 3.0E-26 | 25.52 | 0.008 |
| 2 | rs3131073 | rs141600123 | 28604730 | McKay | Squamous cell lung carcinoma | OR2J3, OR2J2 | AL645937.4 | rs141600123-T | nc_exon | 0.098 | 3.0E-11 | 10.52 | 1.244 |
| 2 | rs3131073 | rs145283874 | 29808027 | Tedja | Myopia (age of diagnosis) | NR | OR2J2 - OR2J4P | rs145283874-T | intergenic | 0.102 | 3.0E-08 | 7.52 | 0.063 |
| 2 | rs3131073 | rs116826541 | 28604730 | McKay | Lung cancer | OR2J2, OR14J1 | OR2J2 - OR2J4P | rs116826541-G | intergenic | 0.090 | 4.0E-12 | 11.40 | 1.156 |
| 2 | rs3131073 | rs3130746 | 28928442 | Tian | Tuberculosis | NR | OR2J4P - AL645937.3 | rs3130746-? | intergenic | NR | 3.0E-06 | 5.52 | 0.178 |
| 2 | rs3131073 | rs3130825 | 30048462 | Kim | Heel bone mineral density | - | AL672167.1 | rs3130825-? | intron | NR | 2.0E-09 | 8.70 | 0.021 |
| 2 | rs3131073 | rs3130834 | 29059683 | Michailidou | Breast cancer | NR | AL672167.1 | rs3130834-C | intron | 0.096 | 5.0E-09 | 8.30 | 0.067 |
| 2 | rs3131073 | rs139244745 | 28604730 | McKay | Lung cancer | OR2J2, OR14J1 | AL672167.1 | rs139244745-G | intron | 0.095 | 1.0E-12 | 12.00 | 1.156 |
| 2 | rs3131073 | rs139244745 | 28604730 | McKay | Lung cancer in ever smokers | OR2J2, OR14J1 | AL672167.1 | rs139244745-G | intron | 0.088 | 8.0E-08 | 7.10 | 1.162 |
| 2 | rs3131073 | rs139244745 | 28604730 | McKay | Squamous cell lung carcinoma | OR2J2, OR14J1 | AL672167.1 | rs139244745-G | intron | 0.096 | 3.0E-12 | 11.52 | 1.258 |
| 2 | rs3131073 | rs3129682 | 28240269 | Suhre | Blood protein levels | many | OR14J1 - DDX6P1 | rs3129682-T | intergenic | 0.077 | 1.0E-19 | 19.00 | 0.737 |
| 2 | rs3131073 | rs9257809 | 22961001 | Su | Barrett's esophagus | OR2D12, OR2D13, MHC | OR5V1 | rs9257809-A | intron | 0.870 | 4.0E-09 | 8.40 | 1.210 |
| 2 | rs3131073 | rs9257809 | 27527254 | Gharahkhani | Barrett's esophagus | MHC | OR5V1 | rs9257809-A | intron | - | 6.0E-09 | 8.22 | 1.263 |
| 2 | rs3131073 | rs9257809 | 27527254 | Gharahkhani | Barrett's esophagus or Esophageal adenocarcinoma | MHC | OR5V1 | rs9257809-A | intron | - | 6.0E-09 | 8.22 | 1.230 |
| 2 | rs3131073 | rs9257809 | 29059683 | Michailidou | Breast cancer | NR | OR5V1 | rs9257809-G | intron | 0.100 | 3.0E-08 | 7.52 | 0.063 |
| 2 | rs3131073 | rs9257809 | 26651848 | Rivera | Sarcoidosis (Lofgren's syndrome vs non-Lofgren's syndrome) | OR12D3, OR12D2 | OR5V1 | rs9257809-G | intron | 0.233 | 1.0E-20 | 20.00 | - |
| 2 | rs3131073 | rs1233491 | 25827949 | Kunz | Cutaneous lupus erythematosus | MAS1L | RPS17P1 - AL662860.1 | rs1233491-C | intergenic | 0.088 | 8.0E-10 | 9.10 | 2.200 |
| 2 | rs3131073 | rs1233491 | 26651848 | Rivera | Sarcoidosis (Lofgren's syndrome vs non-Lofgren's syndrome) | MAS1L, LOC729653 | RPS17P1 - AL662860.1 | rs1233491-C | intergenic | 0.230 | 1.0E-20 | 20.00 | - |
| 2 | rs3131073 | rs1233480 | 29059683 | Michailidou | Breast cancer | NR | AL662860.1 | rs1233480-T | intron | 0.110 | 5.0E-08 | 7.30 | 0.059 |
| 2 | rs3131073 | rs385816 | 28928442 | Tian | Pneumonia | NR | AL662860.1 - LINC01015 | rs385816-? | intergenic | NR | 5.0E-07 | 6.30 | 0.074 |
| 2 | rs3131073 | rs926552 | 27618448 | Liu | Diastolic blood pressure | SNORD32B | GABBR1 | rs926552-T | intron | 0.111 | 7.0E-08 | 7.15 | 0.260 |
| 2 | rs3131073 | rs115870917 | 28604730 | McKay | Lung cancer | GABBR1, MOG | SUMO2P1 - MOG | rs115870917-C | regulatory | 0.088 | 7.0E-19 | 18.15 | 1.198 |
| 2 | rs3131073 | rs3131856 | 28928442 | Tian | Tuberculosis | NR | SUMO2P1 - MOG | rs3131856-? | regulatory | NR | 1.0E-07 | 7.00 | 0.195 |
| 2 | rs3131073 | rs3116813 | 29059683 | Michailidou | Breast cancer | NR | ZFP57 - ZDHHC20P1 | rs3116813-A | intergenic | 0.103 | 6.0E-07 | 6.22 | 0.055 |
| 2 | rs3131073 | rs147680653 | 27197191 | Fehringer | Cancer (pleiotropy) | Intergenic | AL645939.3 - HLA-V | rs147680653-G | regulatory | NR | 1.0E-06 | 6.00 | - |
| 2 | rs3131073 | rs147680653 | 28604730 | McKay | Lung cancer | IFITM4P, HCG4 | AL645939.3 - HLA-V | rs147680653-G | regulatory | 0.083 | 7.0E-19 | 18.15 | 1.219 |
| 2 | rs3131073 | rs147680653 | 28604730 | McKay | Squamous cell lung carcinoma | IFITM4P, HCG4 | AL645939.3 - HLA-V | rs147680653-G | regulatory | 0.082 | 1.0E-14 | 14.00 | 1.322 |
| 2 | rs3131073 | rs9258375 | 28928442 | Tian | Urinary tract infection frequency | NR | AL645939.3 - HLA-V | rs9258375-? | regulatory | NR | 8.0E-06 | 5.10 | 0.057 |
| 3 | rs2189373 | rs115242751 | 28540026 | Anney | Autism spectrum disorder or schizophrenia | many | TRIM10 | rs115242751-T | intron | - | 1.0E-16 | 16.00 | 1.170 |
| 3 | rs2189373 | rs2523722 | 22883433 | Irish | Schizophrenia | MHC, TRIM26 | TRIM26 | rs2523722-G | intron | 0.737 | 1.0E-16 | 16.00 | 1.250 |
| 3 | rs2189373 | rs116408368 | 28540026 | Anney | Autism spectrum disorder or schizophrenia | many | TRIM26 | rs116408368-T | intron | - | 7.0E-17 | 16.15 | 1.150 |
| 3 | rs2189373 | rs116408368 | 30285260 | Ikeda | Schizophrenia | TRIM26 | TRIM26 | rs116408368-T | intron | NR | 1.0E-21 | 21.00 | 1.203 |
| 3 | rs2189373 | rs2021722 | 21926974 | Ripke | Schizophrenia | TRIM26 | TRIM26 | rs2021722-C | intron | 0.780 | 2.0E-12 | 11.70 | 1.150 |
| 3 | rs2189373 | rs2021722 | 23453885 | Smoller | Autism spectrum disorder, attention deficit-hyperactivity disorder, bipolar disorder, major depressive disorder, and schizophrenia (combined) | MHC region | TRIM26 | rs2021722-? | intron | 0.789 | 2.0E-12 | 11.70 | - |
| 3 | rs2189373 | rs885916 | 29844566 | Davies | General cognitive ability | HCG17 | HCG17 | rs885916-T | intron | NR | 1.0E-11 | 11.00 | 6.793 |
| 3 | rs2189373 | rs2189373 | 29844566 | Davies | General cognitive ability | HCG17 | HCG17 | rs2189373-T | intron | NR | 5.0E-09 | 8.30 | 5.835 |
