# Supplementary material for: Phenotypic and genetic analysis of a wellbeing factor score in the UK Biobank and the impact of childhood maltreatment and psychiatric illness
Source: Transl Psychiatry. 2022 Mar 19;12:113. doi: 10.1038/s41398-022-01874-5 (PMC8933416; doi:10.1038/s41398-022-01874-5)
Supplement: Supplementary file 6 — Supplementary Information [file 41398_2022_1874_MOESM6_ESM.pdf]

Supplemental Information

Phenotypic and genetic analysis of a wellbeing factor score in the UK Biobank and the impact of childhood maltreatment and psychiatric illness

Javad Jamshidi (MSc.),[1,2] Peter R Schofield (DSc. PhD.),[1,3] Justine M Gatt (PhD.),[1,2]^ & Janice M Fullerton (PhD.)[1,3]^* (^joint senior authors)

1. Neuroscience Research Australia, Sydney, NSW, Australia
2. School of Psychology, University of New South Wales, Sydney, NSW, Australia
3. School of Medical Sciences, University of New South Wales, Sydney, NSW, Australia

***Corresponding author:** Associate Professor Janice M Fullerton; Phone (+61 2 93991836);

Postal address: Neuroscience Research Australia, Barker St, Randwick Sydney NSW 2031 Australia.

E-mail address: j.fullerton@neura.edu.au

Contents

Supplementary Methods 3
Figure S1. Schematic of analysis workflow for the current study.......... 7
Supplementary Results 8
Figure S2. Scree plot of the principal component analysis. 8
Figure S3. Distribution of wellbeing index score. 8
Figure S4. Regional plots of five loci which exceeded genome-wide significance for the wellbeing index score in discovery sample. 9
Figure S5. MAGMA tissue specific enrichment using 53 specific tissue types for the wellbeing index GWAS. 11
Figure S6. Manhattan plots for GWAS of wellbeing core subcomponents, the negative indicators of mental health and childhood maltreatment. 12
Figure S7. SNP-based heritability of the wellbeing index score and its subcomponents. 15
Figure S8. Effect of frequency of exposure to different childhood maltreatment types on wellbeing index score. 16
Figure S9. Regression analysis on exposure to different types of childhood maltreatment (as binary exposed/not-exposed variables) on the wellbeing index. 17
Figure S10. The effect of a single type of maltreatment exposure compared to no maltreatment exposure on mean wellbeing index. 18
Figure S11. Genetic correlation (r_g) between the childhood maltreatment sum score GWAS and the wellbeing index, its subcomponents, and negative mental health traits in the UK Biobank. 19
Figure S12. Distribution of wellbeing index scores within each category of self-reported psychiatric diagnoses in the discovery cohort. 20
Supplementary References 21

Defining the wellbeing phenotype

We first reviewed all items in the baseline questionnaire (issued in 2006-2010) and the follow-up mental health questionnaire (issued from 2016) to identify those self-report questions that provide some measurement of subjective or psychological wellbeing. Due to the time-lag between the baseline and follow-up questionnaires, and the smaller number of wellbeing-related questions and participants who completed follow-up assessment, we focused on relevant items from the baseline questionnaire – which focused only on subjective elements of wellbeing. There was only one question "To what extent do you feel your life to be meaningful?" (Data-Field 20460) related to psychological wellbeing which was in the follow-up questionnaire, and we did not use in the current study.

We identified six items in the baseline dataset that related to the 'core' concept of wellbeing:

a) "In general, how happy are you?",

plus five questions on different domains of life satisfaction – "In general, how satisfied are you with your…"

b) …family relationships,

c) …friendships,

d)…financial situation,

e)…health,

f)…work that you do;

These questions (Data-Fields 4526, 4559, 4570, 4581, 4548, 4537 respectively) were rated on a 6-point Likert scale (e.g., from 'extremely happy' to 'extremely unhappy'). Participants with "Do not know" or "Prefer not to answer" were coded as missing.

While job satisfaction is also an important aspect of wellbeing, ~29% of individuals at baseline had answered "I am not employed" – thus, to maximise the sample size and avoid circumstantial bias in the wellbeing score relating to employment status, the variable job satisfaction was excluded from factor analysis. Items were reverse scored so that higher scores corresponded to higher happiness/satisfaction.

Principal component analysis was performed in SPSS v25 for five subjective wellbeing items and a factor score created which we herein refer to as the "wellbeing index" (Figure S2). Participants who had missing data for any of the five core wellbeing questions were excluded. To evaluate the goodness of fit for this factor model, we employed a confirmatory factor analysis in the validation dataset using *lavvan* version 0.6.7 package in R.

Independent confirmatory sample

Participants in this sample were selected from those who completed the same baseline questionnaire during the imaging visit (after 2014), were not in our discovery sample, and had no missing data for any of the five items used to derive the wellbeing index (happiness, friendship, family, financial and health satisfaction). Further we identified and removed any participants who were genetically related to participants in the discovery sample (up to the third degree relative, IBD kinship coefficients >0.044), leaving N=23,703 participants in this independent confirmatory sample.

Negative indicators of mental health - coding

For *loneliness*, the question “Do you often feel lonely?” (Data-Field 2020), was answered with a binary “yes” or “no” response.

The *neuroticism* measure was a summary score based on 12 neurotic behaviour domains (Data-Field 20127), with a score of 0-12 [1]. These 12 questions are from the Eysenck Personality Inventory Neuroticism scale (EPIN-R) [2]. The questions are: "Does your mood often go up and down?"; "Do you ever feel 'just miserable' for no reason?"; "Are you an irritable person?”; "Are your feelings easily hurt?"; "Do you often feel 'fed-up'?"; "Would you call yourself a nervous person?"; "Are you a worrier?"; "Would you call yourself tense or 'highly strung'?"; "Do you worry too long after an embarrassing experience?"; "Do you suffer from 'nerves'?"; "Do you often feel lonely?"; "Are you often troubled by feelings of guilt?"

A binary variable was constructed using two lifetime *depressive symptom* questions – “have you ever had a time when you were feeling depressed or down for at least a whole week?" and "Have you ever had a time when you were uninterested in things or unable to enjoy the things you used to for at least a whole week?" (Data-Fields 4598 and 4631, respectively) – participants who answered yes to one or both questions were coded as “yes” for depressive symptoms, and participants who answered no to both were coded as “no”. For the *“seen GP or psychiatrist”* binary variable, participants who answered yes to either of the two questions – "Have you ever seen a psychiatrist for nerves, anxiety, tension or depression?" and "Have you ever seen a general practitioner (GP) for nerves, anxiety, tension or depression?" (Data-Fields 2100 and 2090, respectively) – were coded as “yes”, and those who answered no to both were coded as “no”.

Childhood maltreatment

There are five items that relate to childhood maltreatment in the UK Biobank Mental Health Questionnaire, which were derived from the Childhood Trauma Questionnaire [3] which was part of

the follow-up mental health assessment which was completed from 2016; (Data-Fields 20489-20491 respectively). The items are: "When I was growing up...

a) …I felt loved **(Emotional neglect)**
b) …people in my family hit me so hard that it left me with bruises or marks **(Physical abuse)**
c) …I felt that someone in my family hated me **(Emotional abuse)**
d) …someone molested me (sexually) **(Sexual abuse)**
e) …there was someone to take me to the doctor if I needed it **(Physical neglect)**

To evaluate the accumulative effect, each maltreatment type was dichotomised from a 5-point Likert response ("Never true" to "very often true") to a binary trauma "exposed" or "not-exposed" variable, and a *sum score* was created by summing across the first four maltreatment types (ranging from 0 to 4). For sexual, physical, and emotional abuse, all responses except "never true", were considered as trauma exposed. For emotional neglect, all responses except "very often true" and "often true" were considered trauma exposed [see Table S2 for details], consistent with a higher cut-off for emotional neglect within the Child Trauma Questionnaire [3].

The physical neglect category ("...there was someone to take me to the doctor if I needed it") (Data-Field 20491) was not included in the maltreatment sum score after analysis of the wellbeing index against the categorical responses indicating a potential misunderstanding of this question (Data-Field 20491). The people who answered "never" (N=893, mean=0.053) were more numerous than those who answered "rarely" (N=317) and had a significantly higher wellbeing index score than both the population mean and the mean value of individual "exposed" subcategories (Figure S8.e), suggesting that some participants may have responded "never" if they didn't need to be taken to the doctor (i.e., they had no ailments), rather than not having someone to take them.
There were 45,723 participants with non-missing childhood maltreatment sum score and wellbeing index for analysis.

Effect of childhood maltreatment on the wellbeing index score

First, we examined the impact of each individual type of childhood maltreatment on the wellbeing index to investigate their effects as categorical variables relating to the frequency of the maltreatment experience. Then, the cumulative impact was investigated using maltreatment sum score. Mean differences across all categories were tested using Kruskal-Wallis test, and mean differences between two adjacent groups were tested using Wilcoxon test. Further, linear regression models adjusting for age, age-squared, sex and Townsend Deprivation Index [Data-Field 189] were

used to test how different types of childhood maltreatment (or the sum of exposures across multiple types of maltreatment) can influence the wellbeing index in the presence of possible confounding. Standardised estimates from the linear models were considered as the magnitude of the effect of the dependant variable. To investigate the unique effect of each type of maltreatment, a subset of participants who reported a single childhood maltreatment type were compared to those without any type of maltreatment, for mean wellbeing index score differences.

Functional Mapping and Annotation of GWAS loci

Functional Mapping and Annotation of Genome-Wide Association Studies (FUMA) was used for the wellbeing GWAS annotation [4]. FUMA first identified linkage disequilibrium (LD) independent SNPs with genome-wide significant association (r2<0.6, $p<1\times10^{-8}$) as independent significant SNPs, then defined independent lead SNPs as those with $p<1x10^{-8}$ and LD $r^2<0.1$. The LD blocks of independent lead SNPs were merged into one genomic locus if they were closer than 250kb. All SNPs (with maximum p<0.05) with r2≥0.6 with any of the independent significant SNPs were included for further annotation. We used UK Biobank release 2bB reference panel for all the analysis, except for annotation of the genome -wide significant loci on chromosome 14 (rs79167904), an insertion-deletion variant which was excluded from the UK Biobank release 2bB reference panel. Instead, the smaller 1000 Genome Phase3 reference panel, which included indel variants was used to annotate rs79167904.

FUMA uses three strategies to map SNPs to genes: 1) Positional mapping, based on physical proximity of a SNP to a gene; 2) expression quantitative trait loci (eQTL) mapping, to identify genes whose transcript expression is significantly associated with allelic variation of the SNP; and 3) chromatin interaction mapping, which links SNPs to genes based on significant chromatin interactions between each SNP's genomic region and nearby or distant genes. For positional mapping, we used a 10kb window, without any other filters. For eQTL mapping, only databases employing brain tissue were selected - PsychENCODE [5], xQTLServer [6], CommonMind Consortium [7], BRAINEAC [8], and GTEx brain tissues V6-8 [9] - and only significant SNP-gene pairs with FDR<0.05 were used. For chromatin interaction mapping, we included PsychENCODE and brain tissues/cell types from Hi-C chromatin interaction dataset and set an FDR threshold of $p<1\times10^{-6}$ to define significant interactions.

Tissue specific enrichment of genes mapped to the wellbeing index GWAS signals, employing the full distribution of SNP p-values, was performed in MAGMA [10] (implemented in FUMA) using GTEx v8 differentially expressed gene data set for 54 tissue types.

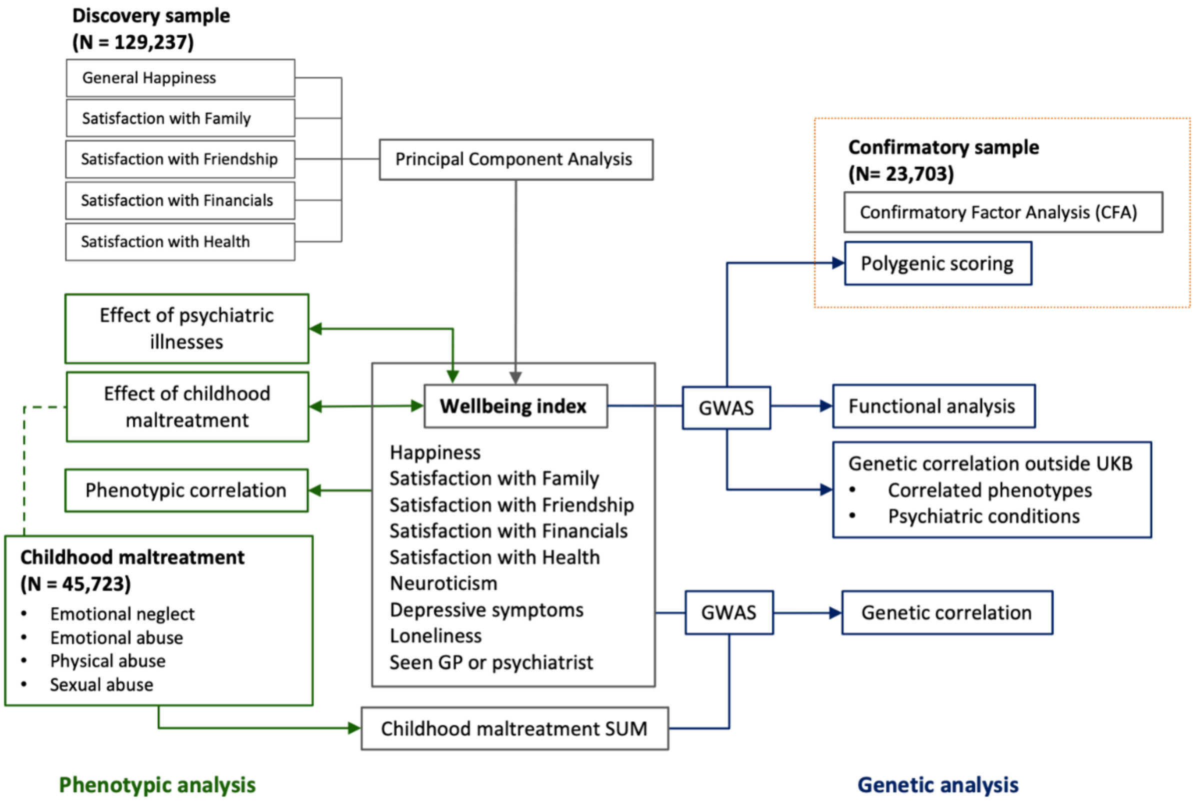

Figure S1. Schematic of analysis workflow for the current study.

Supplementary Results

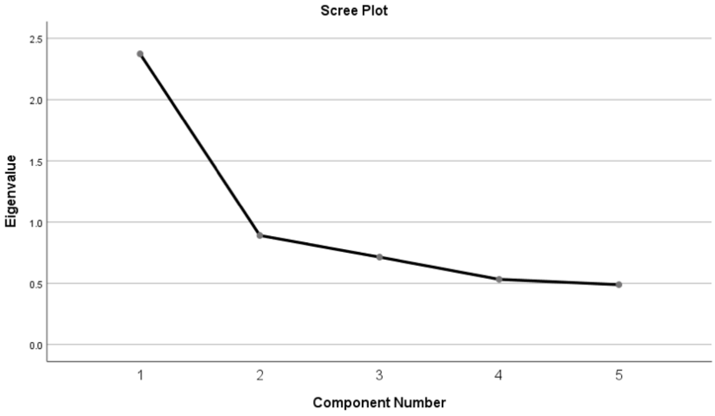

Figure S2. Scree plot of the principal component analysis. Scree plot showing only the first component had an eigenvalue >1. The variance explained by the 5 identified components were 47.476%, 17.807%, 14.279%, 10.651%, and 9.787%, respectively. The wellbeing index factor solution loading of component 1 of component matrix on the individual wellbeing items were: happiness=0.779, family=0.748, friendship=0.726, financial=0.602, health=0.564.

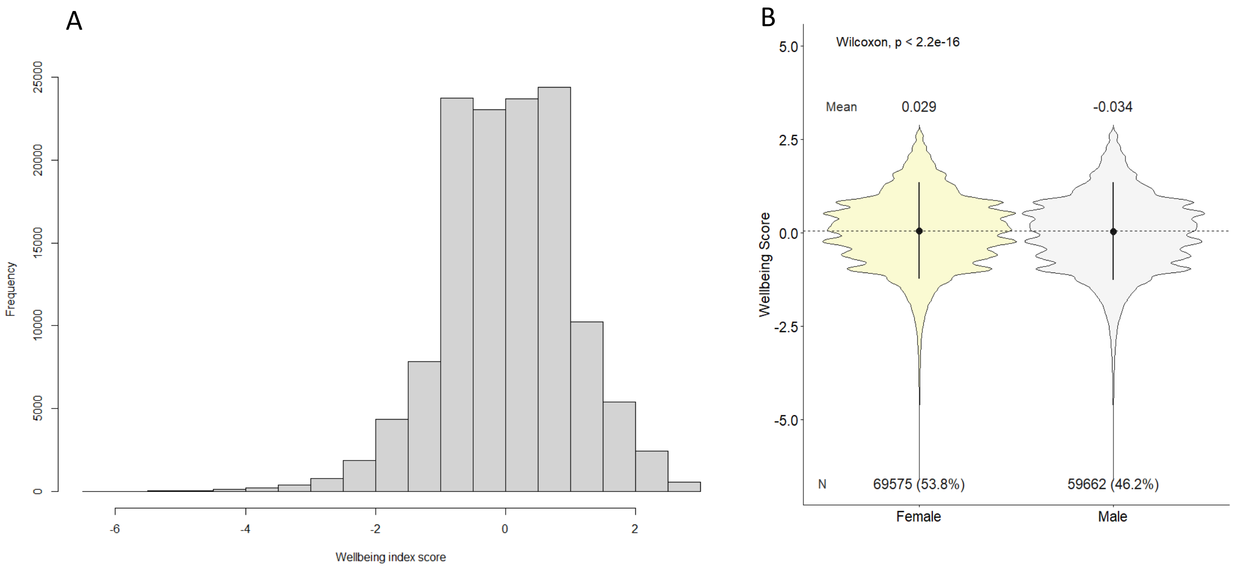

Figure S3. Distribution of wellbeing index score. (A) Histogram of wellbeing index in the whole discovery sample (skewness=-0.373, Kurtosis=4.119, range= -6.338 to 2.603, mean=0, standard deviation=1). (B) Gender-stratified violin plots showing females with a slightly higher mean wellbeing index score than males (0.029 vs -0.034 respectively; Wilcoxon test $p<2.2e^{-16}$).

Figure S4. Regional plots of five loci which exceeded genome-wide significance for the wellbeing index score in discovery sample. Plots were generated in FUMA [4].

a) locus 1

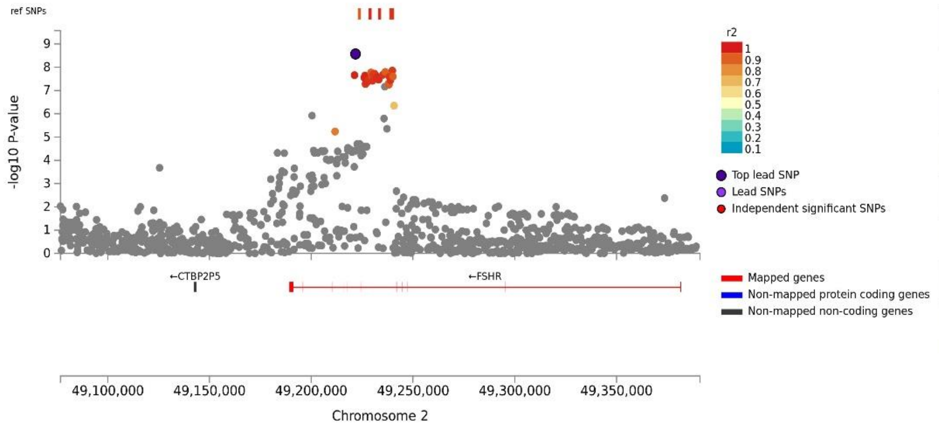

top lead SNP	rs373377070
Chrom	2
BP	49221769
P-value	2.7e-09
#Ind. Sig. SNPs	1
#lead SNPs	1
SNPs within LD	41
GWAS SNPs within LD	35

b) Locus 2

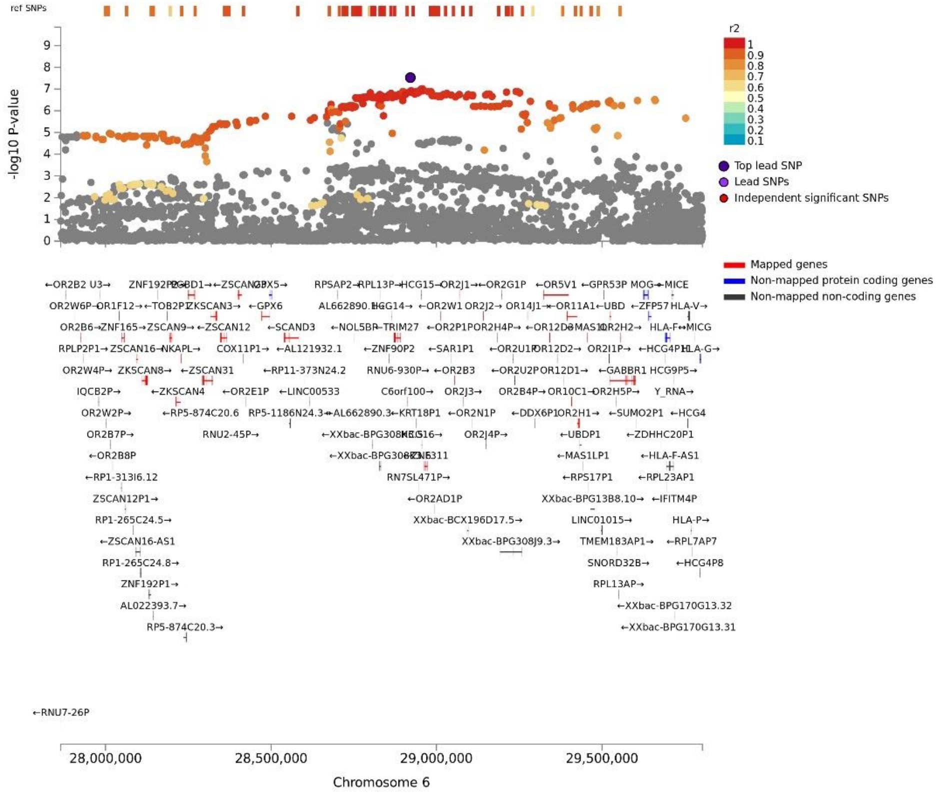

top lead SNP	rs3131073
Chrom	6
BP	28920972
P-value	3e-08
#Ind. Sig. SNPs	1
#lead SNPs	1
SNPs within LD	437
GWAS SNPs within LD	369

c) Locus 3

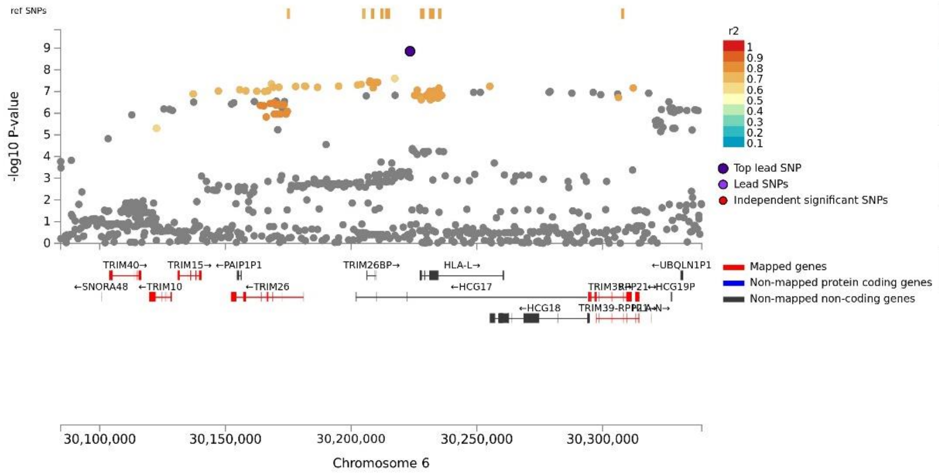

top lead SNP	rs2189373
Chrom	6
BP	30223428
P-value	1.4e-09
#Ind. Sig. SNPs	1
#lead SNPs	1
SNPs within LD	104
GWAS SNPs within LD	90

d) Locus 4

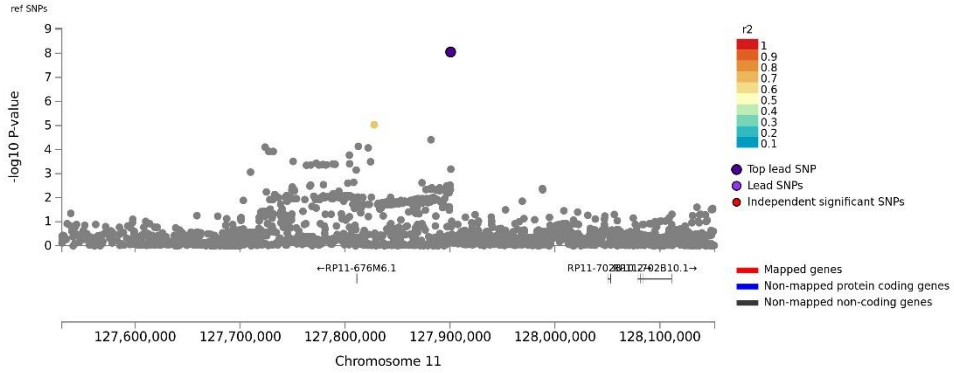

top lead SNP	rs1785039
Chrom	11
BP	127900579
P-value	8.8e-09
#Ind. Sig. SNPs	1
#lead SNPs	1
SNPs within LD	2
GWAS SNPs within LD	2

e) Locus 5

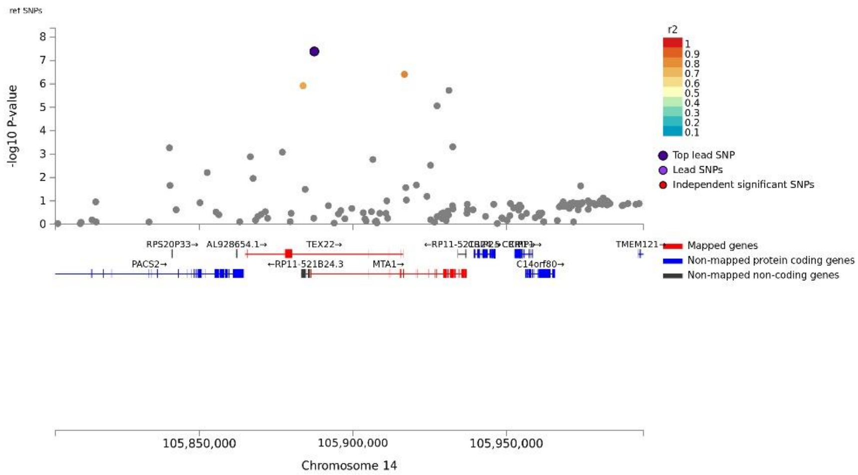

top lead SNP	rs79167904
Chrom	14
BP	105887452
P-value	4.1e-08
#Ind. Sig. SNPs	1
#lead SNPs	1
SNPs within LD	3
GWAS SNPs within LD	3

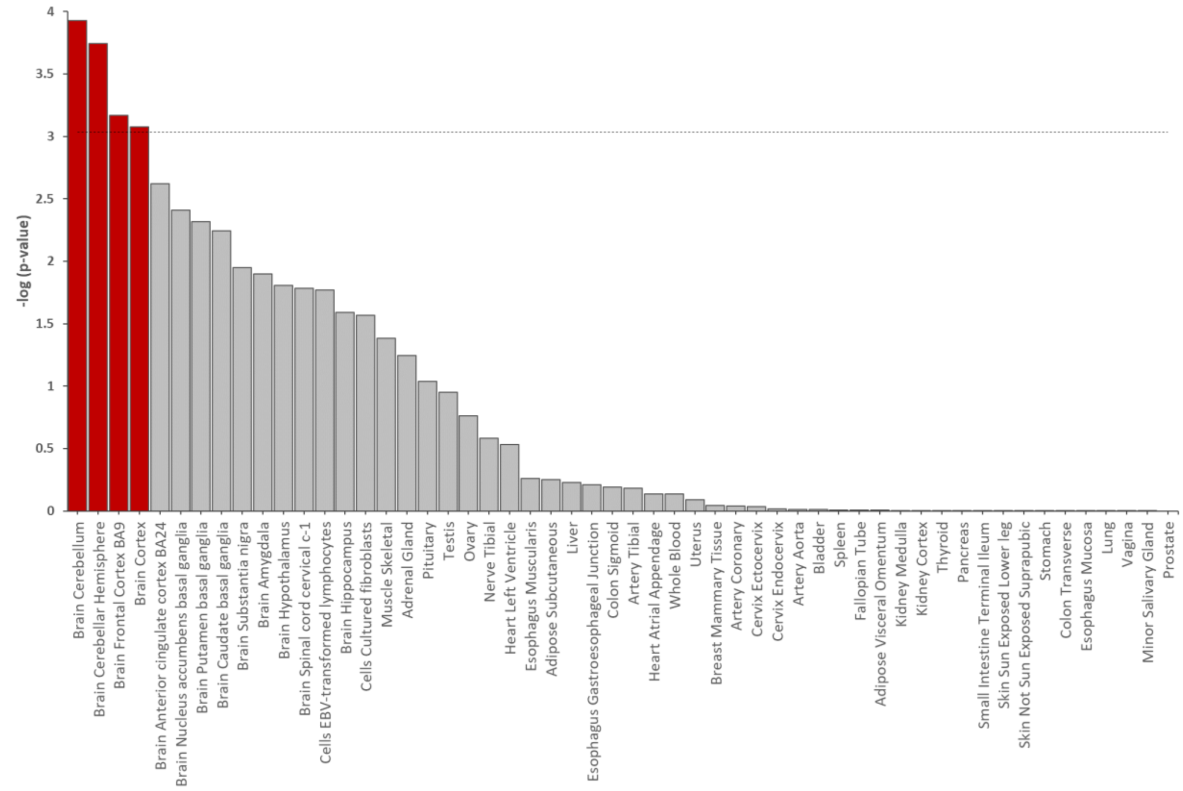

Figure S5. MAGMA tissue specific enrichment using 53 specific tissue types for the wellbeing index GWAS. Bars in red above the dashed line are significantly enriched after Bonferroni correction. The x-axis shows the 53 different tissue categories, and the y-axis shows the –log^{10} P value.

Figure S6. Manhattan plots for GWAS of wellbeing core subcomponents, the negative indicators of mental health and childhood maltreatment. Manhattan plots representing association at 8,068,119 SNPs across the genome, with chromosome and base pair position is on x-axis and negative logarithm of the p-value from infinitesimal model is on y-axis. The red line indicates the genome-wide significance threshold of $p<5\times10^{-8}$. For of loneliness, neuroticism, depressive symptoms and 'seen a psychiatrist or GP for nerves, anxiety, tension, or depression' phenotypes the sample size is smaller due to data missingness. For childhood maltreatment the sample is from the whole UKB sample with data of childhood maltreatment. a) happiness, b) friendship satisfaction, c) family satisfaction, d) financial satisfaction, e) health satisfaction, f) neuroticism, g) loneliness, h) depressive symptoms i) seen GP or psychiatrist for nerves, anxiety, tension, or depression, j) childhood maltreatment.

a) Happiness (N=129,237)

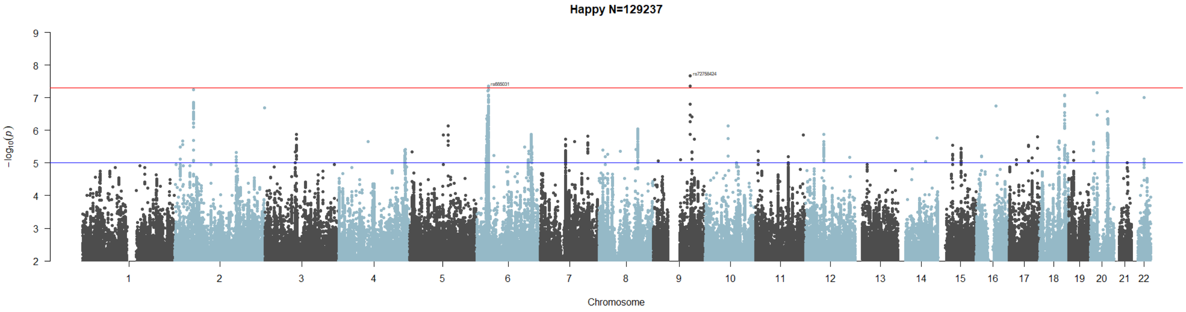

b) Friendship satisfaction (N=129,237)

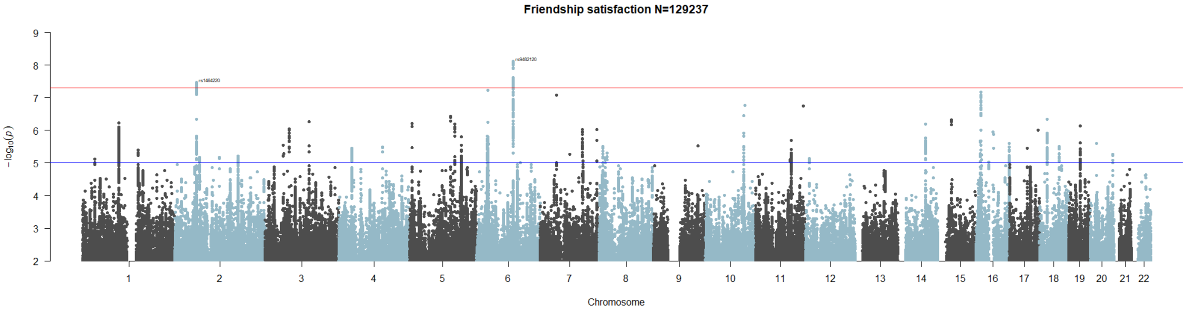

c) Family satisfaction (N=129,237)

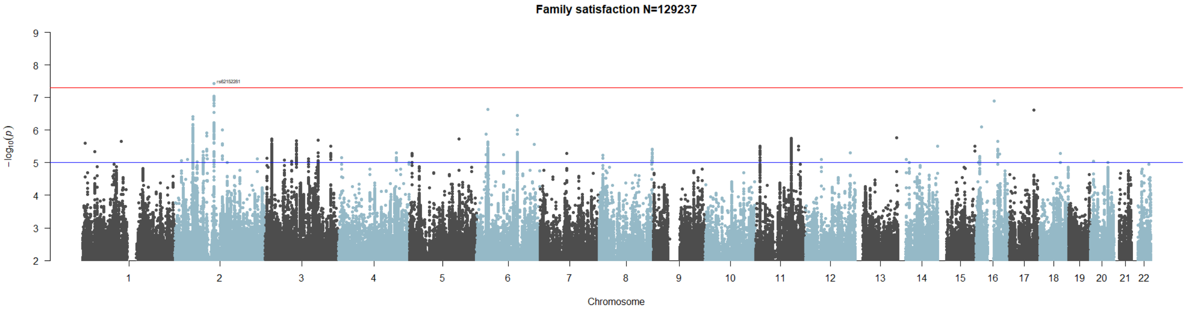

d) Financial satisfaction (N=129,237)

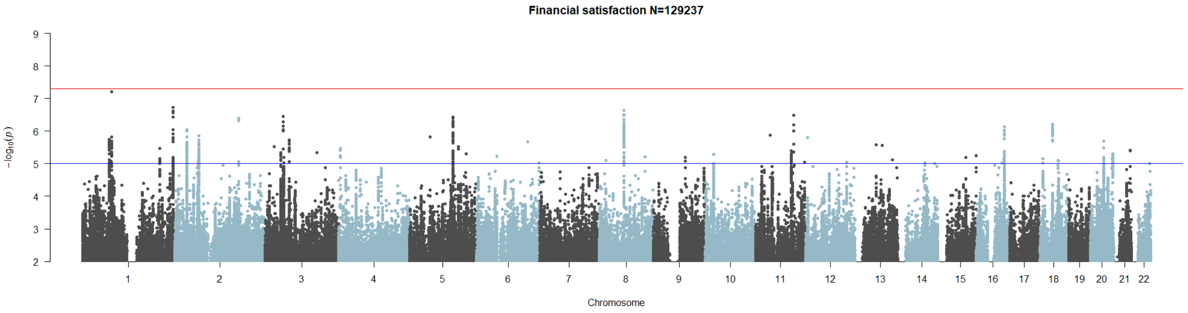

e) Health satisfaction (N=129,237)

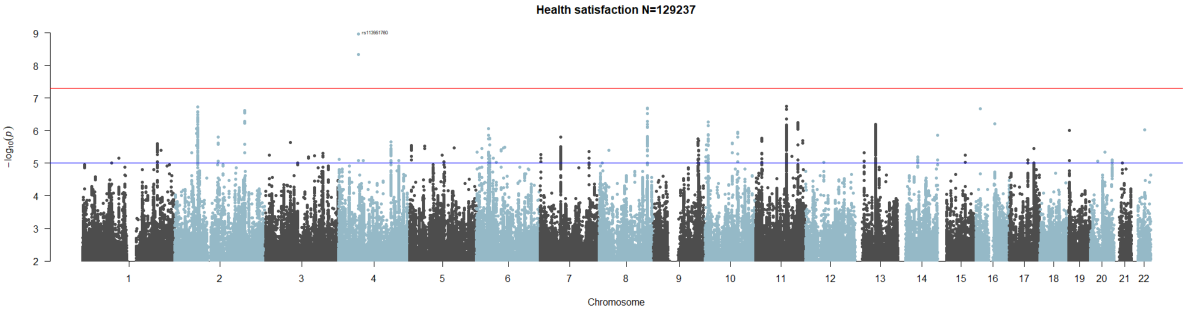

f) Neuroticism (N=107,318)

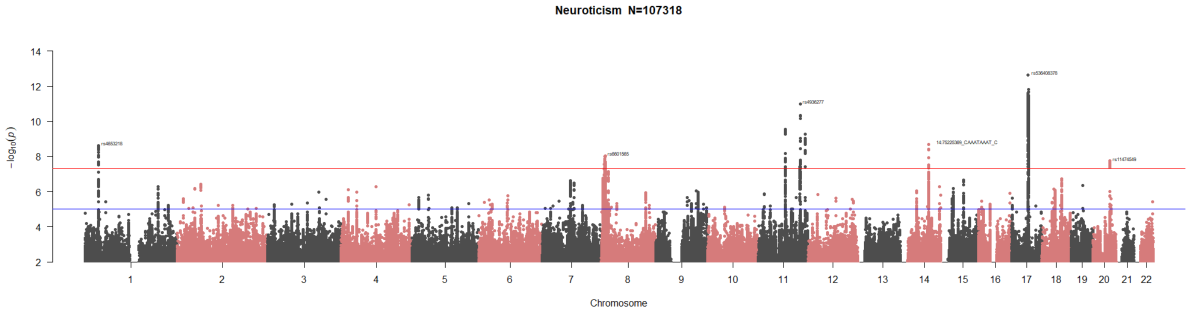

g) Loneliness (N=127,648)

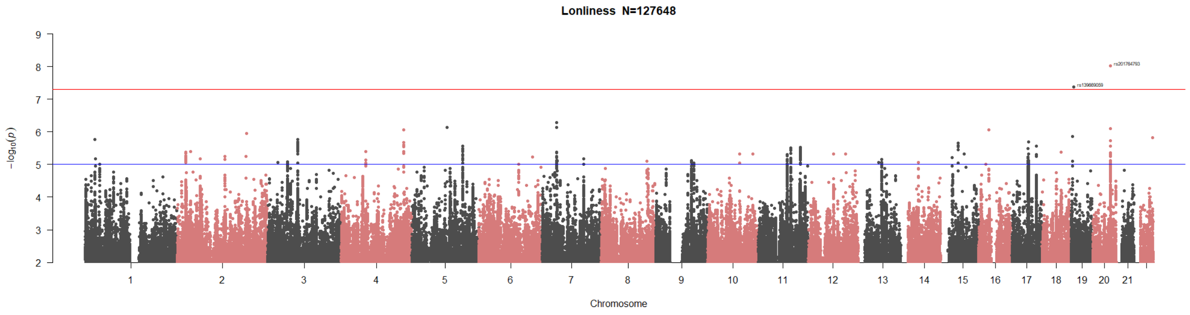

h) Depressive symptoms (N=122,740)

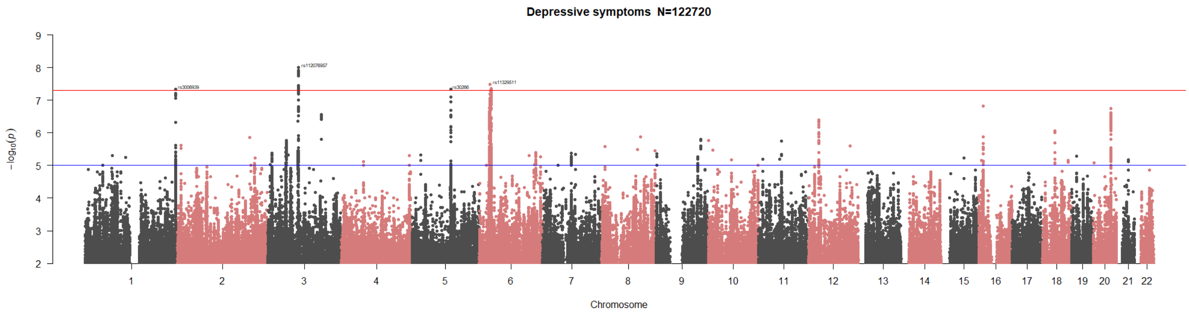

i) Seen GP or psychiatrist for nerves, anxiety, tension, or depression (N=128,221)

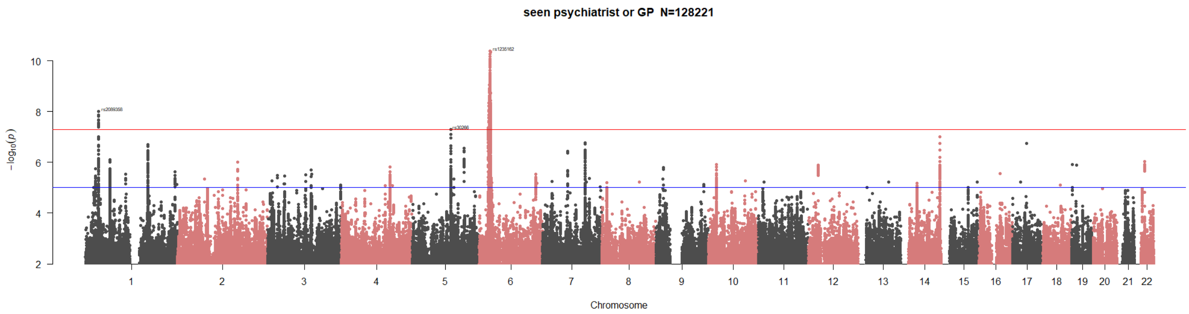

j) Childhood maltreatment (N=129,633)

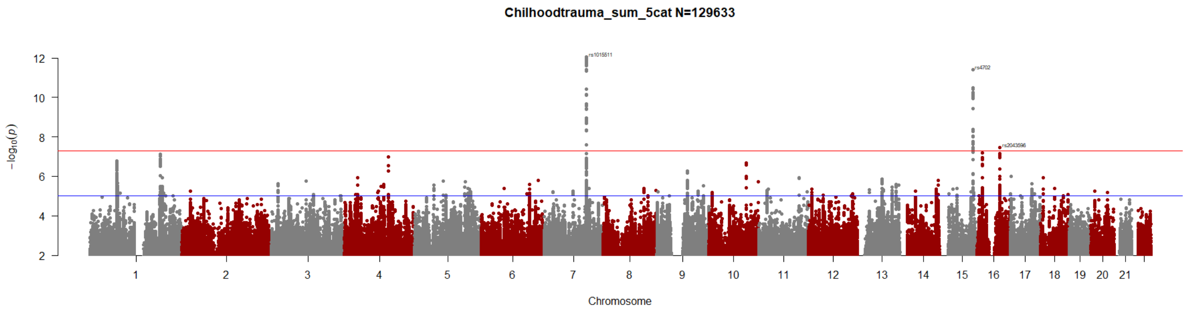

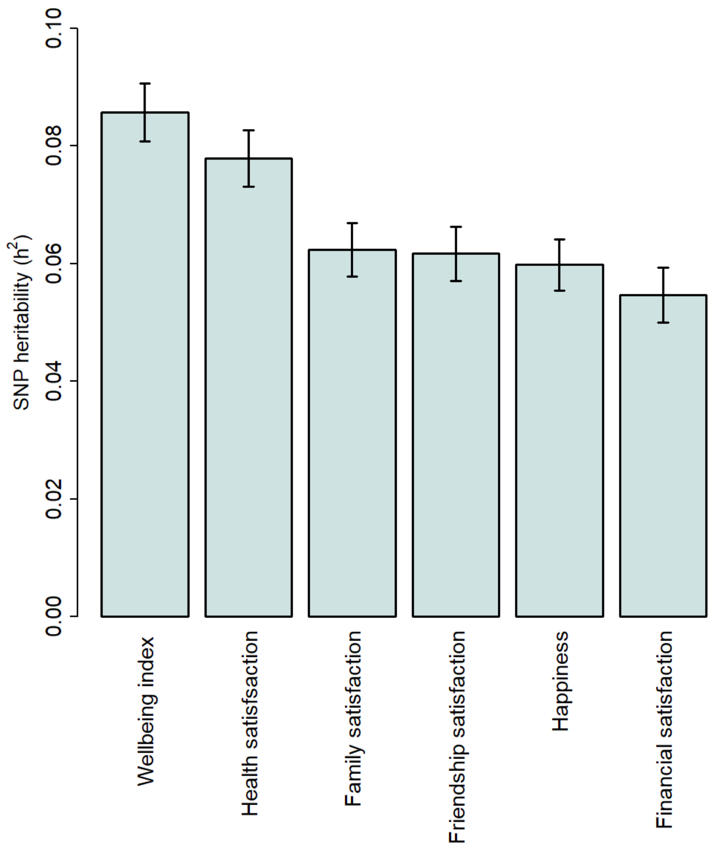

Figure S7. SNP-based heritability of the wellbeing index score and its subcomponents. Derived from the summary statistics of GWAS on 129,237 participants in the discovery sample using Linkage Disequilibrium Score Regression (LDSC) [11]. Error bars represent standard errors.

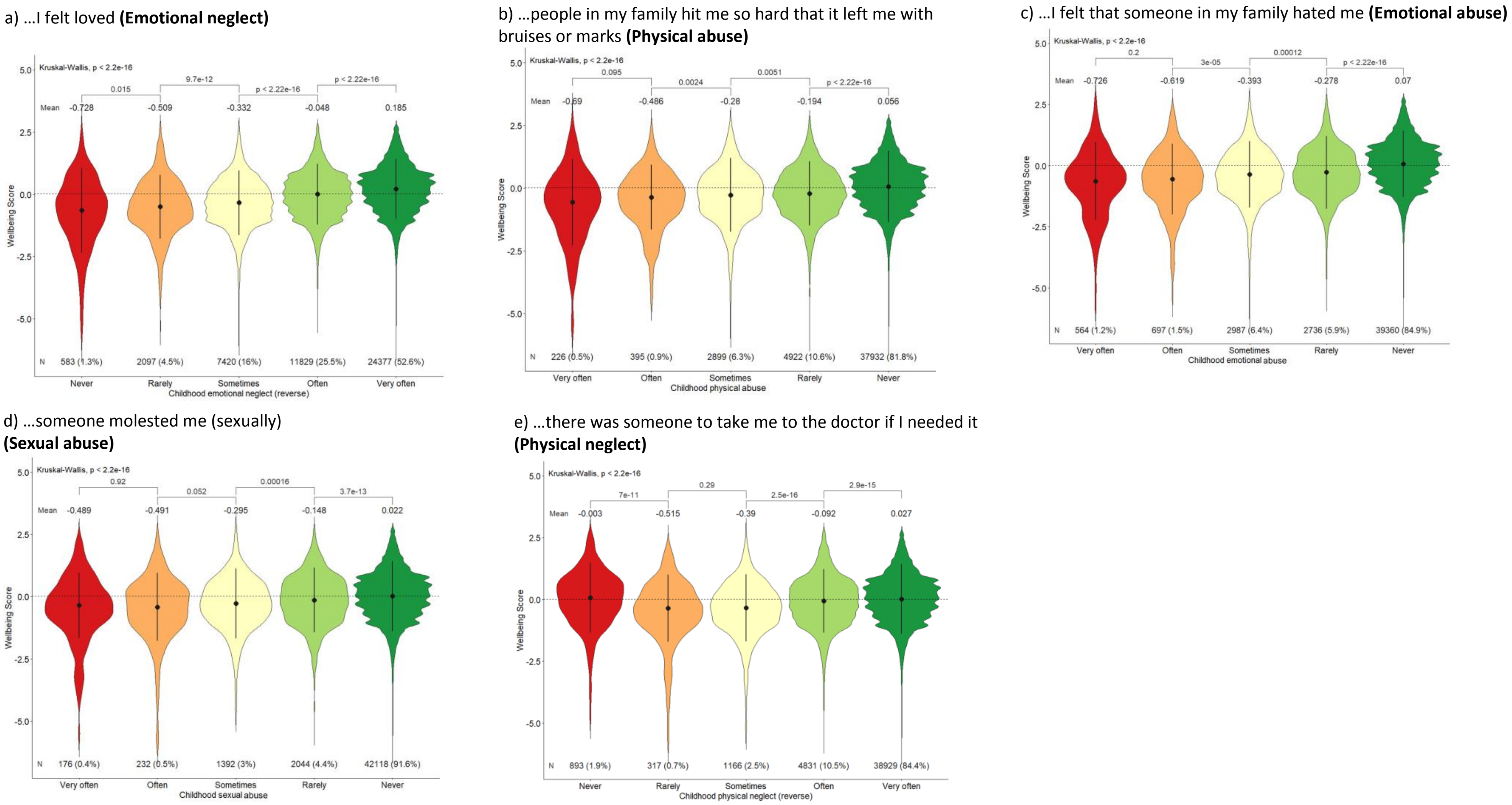

Figure S8. Effect of frequency of exposure to different childhood maltreatment types on wellbeing index score. The mean difference in wellbeing index score across all categories for each maltreatment exposure was tested using Kruskal-Wallis test (all p <$2.2e^{-16}$). In all graphs, the mean wellbeing index z-score for each group category is shown above each violin plot, and the pairwise mean difference between two adjacent groups employed the Wilcoxon test and the p-values are presented. The interquartile range is represented by vertical black lines inside the violin plots, and the dotted horizontal line is the median wellbeing index score in the discovery sample (N=129,237). The number of participants in each group is shown at the bottom of each category.

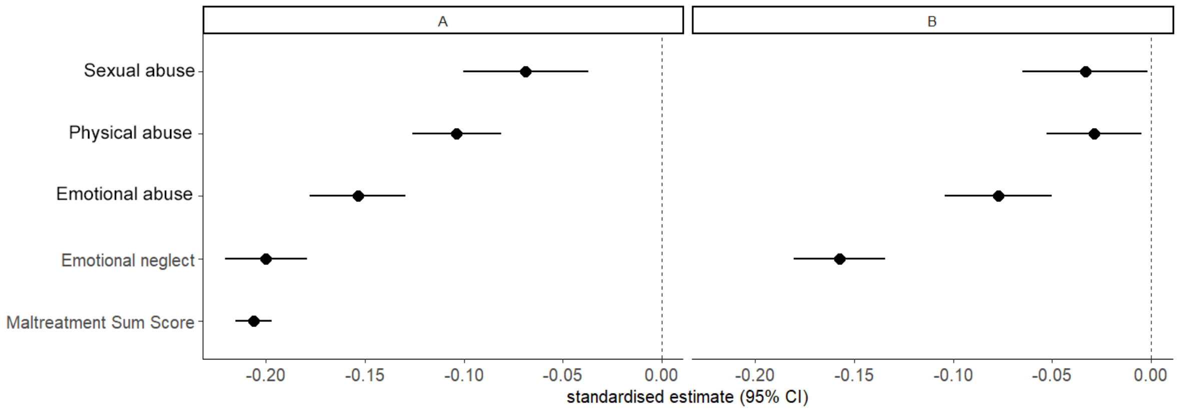

Figure S9. Regression analysis on exposure to different types of childhood maltreatment (as binary exposed/not-exposed variables) on the wellbeing index. Panel A represents the standardised estimates (95% confidence interval) from linear regressions where wellbeing is an outcome and one type of childhood maltreatment (or the sum score) as predictor. Panel B represents the estimates from a multiple linear regression model with all types of maltreatments fitted as predictors in the same model. All regressions are adjusted for age, age-squared, sex and Townsend deprivation index. Maltreatments have been coded as binary variables and the maltreatment sum score represents the accumulation of different types of traumas. Participants represented in each trauma type category are not mutually exclusive, as one person can report multiple types of traumas. The sample size varies slightly across different maltreatment categories due to variable missingness across items (N=45,723 – 46,374).

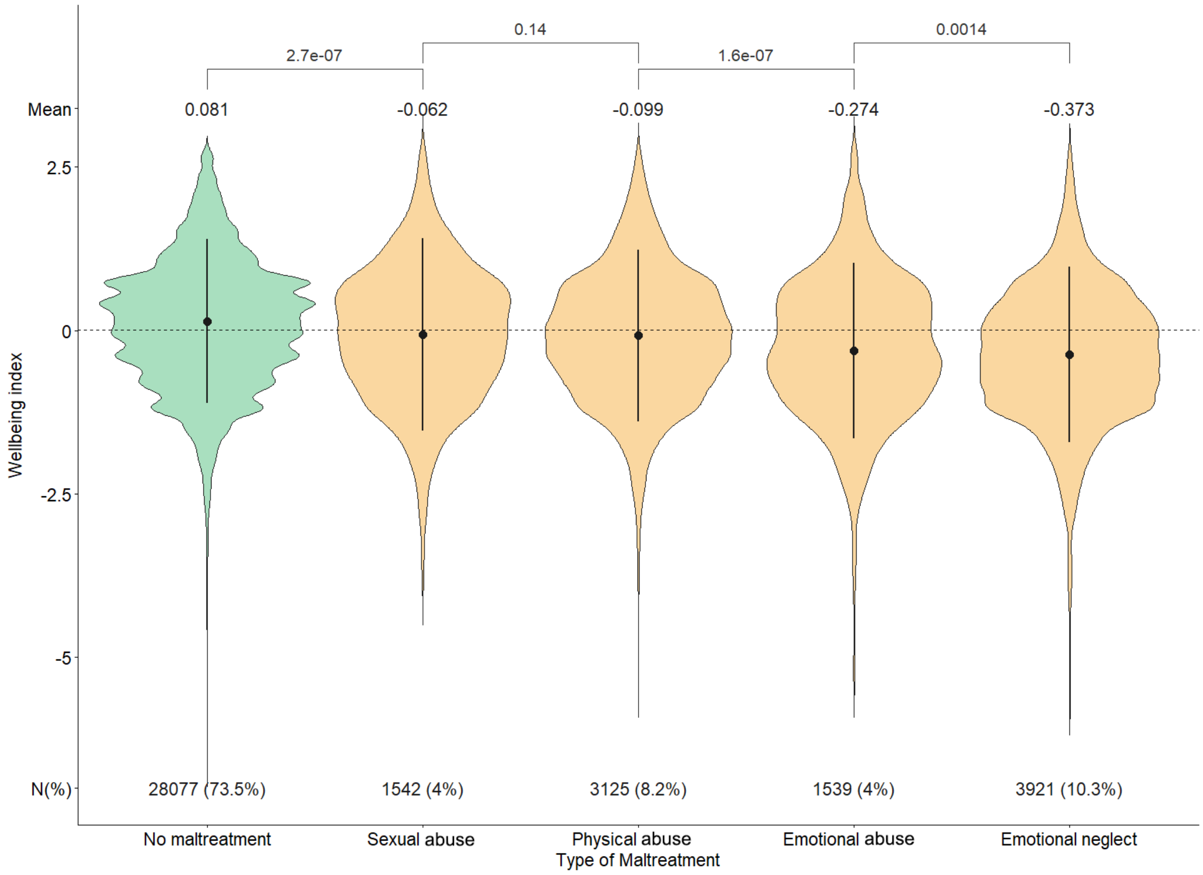

Figure S10. The effect of a single type of maltreatment exposure compared to no maltreatment exposure on mean wellbeing index. Participants with exposure to multiple types of maltreatment have been removed from this analysis. The mean wellbeing index z-score for each group category is shown above each violin plot, and the pairwise mean difference between two adjacent groups employed the Wilcoxon test and the p-values are presented. The interquartile range is represented by vertical black lines inside the violin plots, and the dotted horizontal line is the median wellbeing index score in the sample (N=38,204). The number of participants in each group is shown at the bottom of each category.

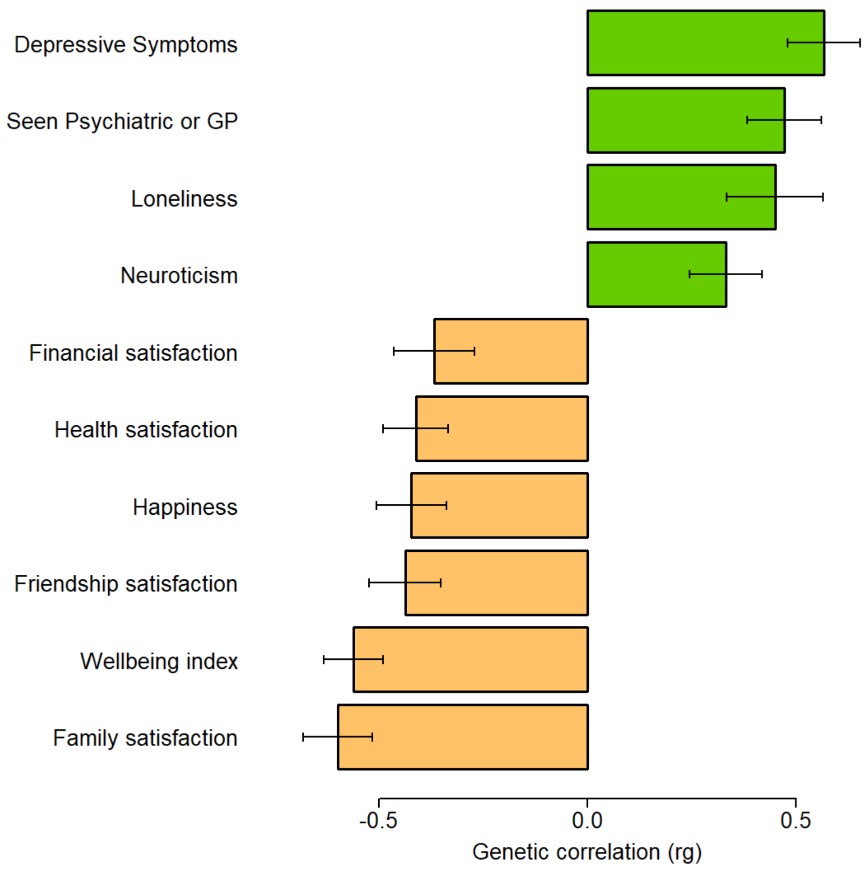

Figure S11. Genetic correlation (r_g) between the childhood maltreatment sum score GWAS and the wellbeing index, its subcomponents, and negative mental health traits in the UK Biobank. Green and orange colouring indicates the positive and negative genetic correlations. All genetic correlations are significant at Bonferroni correction ($p<6.94\times10^{-4}$). Error bars represent 95% confidence intervals.

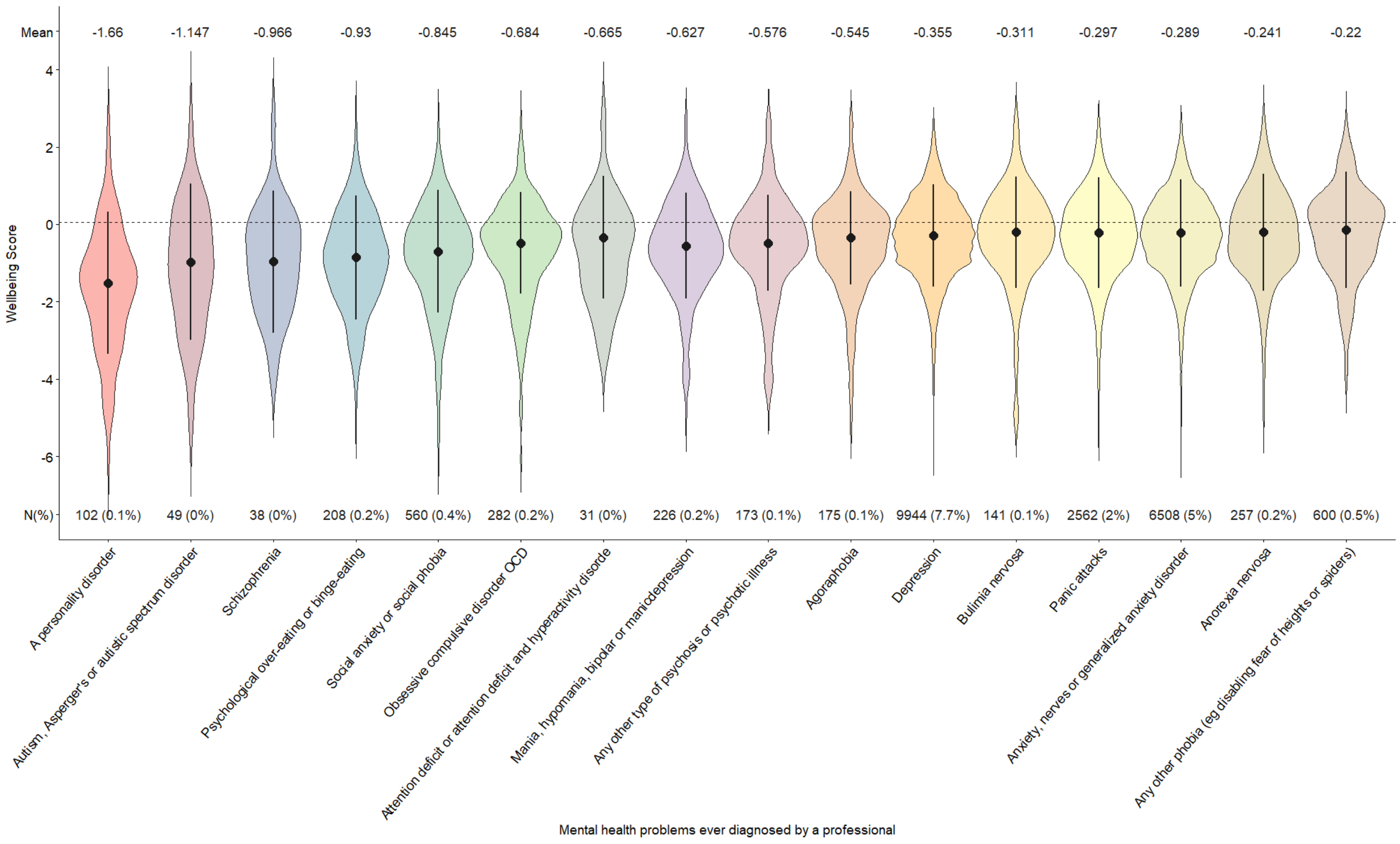

Figure S12. Distribution of wellbeing index scores within each category of self-reported psychiatric diagnoses in the discovery cohort. The number (N) and frequency (%) of participants with each diagnosis is provided along x-axis below the violin plots. The mean wellbeing index score, standardised to a mean of 0 in the general population, is represented by the black dot within plot, and its corresponding numeric value is provided along x-axis above the violin plots for each diagnosis. The interquartile range is represented by vertical black lines inside the violin plots, and the dotted horizontal line is the median wellbeing index score in the discovery sample (N=129,237). Membership in each category was not mutually exclusive, as some participants endorsed receiving more than one lifetime psychiatric diagnosis.

Supplementary References

1. Smith DJ, Nicholl BI, Cullen B, Martin D, Ul-Haq Z, Evans J et al. Prevalence and characteristics of probable major depression and bipolar disorder within UK biobank: cross-sectional study of 172,751 participants. *PLoS One.* 2013; **8:**e75362.
2. Eysenck HJ and Eysenck SBG. *Manual of the Eysenck Personality Questionnaire (junior & adult)*. 1975: Hodder and Stoughton Educational.
3. Bernstein DP, Stein JA, Newcomb MD, Walker E, Pogge D, Ahluvalia T et al. Development and validation of a brief screening version of the Childhood Trauma Questionnaire. *Child Abuse Negl.* 2003; **27:**169-90.
4. Watanabe K, Taskesen E, van Bochoven A, and Posthuma D. Functional mapping and annotation of genetic associations with FUMA. *Nat Commun.* 2017; **8:**1826.
5. Wang D, Liu S, Warrell J, Won H, Shi X, Navarro FCP et al. Comprehensive functional genomic resource and integrative model for the human brain. *Science.* 2018; **362:**
6. Ng B, White CC, Klein HU, Sieberts SK, McCabe C, Patrick E et al. An xQTL map integrates the genetic architecture of the human brain's transcriptome and epigenome. *Nat Neurosci.* 2017; **20:**1418-1426.
7. Fromer M, Roussos P, Sieberts SK, Johnson JS, Kavanagh DH, Perumal TM et al. Gene expression elucidates functional impact of polygenic risk for schizophrenia. *Nat Neurosci.* 2016; **19:**1442-1453.
8. Ramasamy A, Trabzuni D, Guelfi S, Varghese V, Smith C, Walker R et al. Genetic variability in the regulation of gene expression in ten regions of the human brain. *Nat Neurosci.* 2014; **17:**1418-1428.
9. GTEx Consortium. The Genotype-Tissue Expression (GTEx) project. *Nat Genet.* 2013; **45:**580-5.
10. de Leeuw CA, Mooij JM, Heskes T, and Posthuma D. MAGMA: generalized gene-set analysis of GWAS data. *PLoS Comput Biol.* 2015; **11:**e1004219.
11. Bulik-Sullivan BK, Loh PR, Finucane HK, Ripke S, Yang J, Schizophrenia Working Group of the Psychiatric Genomics Consortium et al. LD Score regression distinguishes confounding from polygenicity in genome-wide association studies. *Nat Genet.* 2015; **47:**291-5.
